# What is the impact of e-cigarette adverts on children's perceptions of tobacco smoking? An experimental study

**DOI:** 10.1136/tobaccocontrol-2016-052940

**Published:** 2016-09-05

**Authors:** D C Petrescu, M Vasiljevic, J K Pepper, K M Ribisl, T M Marteau

**Affiliations:** 1 Behaviour and Health Research Unit, Institute of Public Health, University of Cambridge, Cambridge, UK; 2 Department of Health Behaviour, UNC Gillings School of Global Public Health, Cambridge, UK; 3 RTI International, Research Triangle Park, North Carolina, USA; 4 Lineberger Comprehensive Cancer Center, UNC, Chapel Hill, North Carolina, USA

**Keywords:** Electronic nicotine delivery devices, Advertising and Promotion, Priority/special populations

## Abstract

**Objective:**

Exposure to e-cigarette adverts increases children's positive attitudes towards using them. Given the similarity in appearance between e-cigarettes and tobacco cigarettes, we examined whether exposure to e-cigarette adverts has a cross-product impact on perceptions and attitudes towards smoking tobacco cigarettes.

**Methods:**

Children aged 11–16 (n=564) were interviewed in their homes and randomised to one of three groups: two groups saw different sets of 10 images of e-cigarette adverts and one group saw no adverts. Of the 20 e-cigarette adverts, 10 depicted the product as glamorous and 10 depicted it as healthy. The children then self-completed a questionnaire assessing perceived appeal, harms and benefits of smoking tobacco cigarettes.

**Results:**

The analyses were conducted on 411 children who reported never having smoked tobacco cigarettes or used e-cigarettes. Exposure to the adverts had no impact on the appeal or perceived benefits of smoking tobacco cigarettes. While the perceived harm of smoking more than 10 cigarettes per day was similar across groups, those exposed to either set of adverts perceived the harms of smoking one or two tobacco cigarettes occasionally to be lower than those in the control group.

**Conclusions:**

This study provides the first evidence that exposure to e-cigarette adverts might influence children's perceptions of smoking tobacco cigarettes, reducing their perceived harm of occasional smoking. These results suggest the potential for e-cigarette adverts to undermine tobacco control efforts by reducing a potential barrier (ie, beliefs about harm) to occasional smoking.

## Introduction

In countries with comprehensive and established tobacco control policies, fewer adults and children smoke now than several decades ago. As the number of children experimenting with tobacco cigarettes is declining, experimentation with electronic cigarettes (e-cigarettes) is now more common than experimentation with tobacco cigarettes. For example, in England in 2014, 22% of children aged 11–15 had experimented with e-cigarettes while only 18% had experimented with tobacco cigarettes.^1^ Similarly, in the USA e-cigarette use tripled from 2013 to 2014 among high schoolers (5–13%) and middle schoolers (1–4%), whereas tobacco smoking rates declined from 16% to 9% and 4% to 3%, respectively, among these two groups from 2011 to 2014.^2^

The increasing exposure of children to e-cigarette adverts could be contributing to high rates of experimentation with that product.^3^ ^4^ A recent analysis of US panel data shows that promotional spending for e-cigarettes has rapidly increased, reaching $28 million in 2013, over eight times the spending in 2012.^5^ Furthermore, a recent analysis of the online market found that older e-cigarette brands were more likely to sell ‘cigalikes’, claiming they assist smoking cessation and are healthier and cheaper than tobacco cigarettes, while newer brands offered more flavours and were less likely to compare themselves with tobacco cigarettes.^6^ These marketing trends are coupled with American adolescents' exposure to televised e-cigarette adverts increasing by 256% from 2011 to 2013.^7^ Previous studies suggest that such exposure can increase children's positive attitudes and intentions to use e-cigarettes.^8^ Adverts for e-cigarettes that are candy-like or sweet flavoured, as opposed to non-flavoured or tobacco-flavoured, may be especially appealing to adolescents and increase the desire to buy and try these products.^9^

Adverts for e-cigarettes feature messages historically used in the marketing of tobacco cigarettes. For example, e-cigarette adverts often depict their use as glamourous, cool, attractive, liked by celebrities and as a symbol of freedom.^10^ ^11^ In past research, ‘cool’ and ‘glamorous’ characters such as Joe Camel and the Marlboro man increased the appeal and initiation of smoking.^12–15^ E-cigarette adverts may also explicitly or implicitly depict the potential for e-cigarettes to foster health through claims of being safer than tobacco cigarettes.^10^ ^11^ ^16^ Depictions of endorsements by doctors are reminiscent of tobacco cigarette advertising in the 1950s.^17^ These two advertising themes of ‘glamour’ and ‘healthfulness’ are examined in the current study.

Given the physical and motor-behavioural similarities between tobacco cigarettes and many types of e-cigarettes, exposure to e-cigarettes in adverts may affect attitudes towards smoking tobacco cigarettes. Among adult smokers, seeing someone use an e-cigarette in vivo or in video adverts increases the desire and urge to smoke tobacco cigarettes^18^ ^19^ and decreases intentions to abstain from smoking tobacco cigarettes among former smokers.^20^

Two studies have assessed the impact of e-cigarette adverts on the appeal and perceived harm of tobacco smoking among youth, but both studies had important limitations.^8^ ^9^ One used a mixture of adverts with opposing messages, highlighting the differences and similarities between e-cigarettes and tobacco.^8^ Neither study had samples representative of general populations.

The present study builds on the existing limited evidence concerning the possible cross-cueing effects of e-cigarette advertising on the appeal of tobacco smoking among children, assessing the effects of two advertising themes used in earlier tobacco advertising (glamour and healthfulness) and using a sample representative of the general population.

Our primary hypothesis was that exposing children to glamorous e-cigarette adverts increases the appeal of tobacco smoking, and our secondary hypothesis was that exposing children to healthful e-cigarette adverts that emphasise the potential health benefits of e-cigarettes over tobacco cigarettes reduces the appeal of tobacco smoking.

## Methods

### Design

Participants were randomised to one of three groups where they viewed: 10 adverts associating e-cigarette use with glamour, 10 adverts associating e-cigarette use with health or no adverts (control).

### Participants

The study included 564 children aged 11–16. This sample provided 90% power at p=0.01 to detect a medium-size difference in appeal of smoking tobacco cigarettes among the three groups,^21^ allowing for a reduction in sample size caused by excluding children with prior tobacco smoking or e-cigarette use.^1^

### Sampling strategy

A market research agency (ICM Direct; http://www.icm-direct.com/) conducted the interviews. Participants were selected using a random location quota sampling procedure across the UK. Fifty super output areas (SOAs) were randomly selected (with probability of selection proportionate to their size) from the 32 844 lower layer super output areas (LSOAs) in England, 1909 LSOAs in Wales, 2500 data zones in Scotland (equivalent to LSOAs) and 890 LSOAs in Northern Ireland. Recruitment quotas based on census data and Office for National Statistics estimates for age, sex, ethnicity and tenure were set within each LSOA.

### Materials

The adverts used in this study were selected following a pilot study in which participants rated 40 e-cigarette adverts based on how ‘cool’ or ‘healthy’ they portrayed e-cigarettes to be. From the pilot, we selected 10 adverts rated as more glamorous than healthful, showing the largest differences between the two aspects. These ‘glamour’ adverts were reminiscent of tobacco advertising in the 1950–1960s, depicting e-cigarettes as cool, attractive, fashionable, popular and featuring attractive young people. We also selected 10 adverts with the largest differences in ratings of ‘healthy’ over ‘cool’. These adverts featured people wearing white coats and claimed e-cigarettes can aid smoking cessation, do not contain carcinogens found in tobacco cigarettes and are ‘safe and healthy’. Additional by-item analyses showed that the adverts allocated to the glamour group were significantly different and clearly distinct from adverts allocated to the health group (see online supplementary materials).

10.1136/tobaccocontrol-2016-052940.supp1 supplementary materials

### Measures

#### Tobacco cigarette-related measures

*Appeal of smoking tobacco cigarettes* was rated on three bipolar items: unattractive versus attractive, not cool versus cool and boring versus fun.^21^ Responses were recorded on scales ranging from 1 to 5, with higher scores denoting greater appeal. The items formed a reliable scale (α=0.81).

*Perceived positive (pros) and negative (cons) attributes of tobacco smoking* were assessed with the Decisional Balance for Adolescent Smoking Scale developed by Hudmon *et al*^22^ and validated for use with children.^23–25^ Participants rated six items describing perceived pros (eg, ‘Kids who smoke have more friends’) and six items describing perceived cons of smoking (eg, ‘Smoking makes teeth yellow’) on 5-point scales. Scale reliability for both subscales was high (α_pros_=0.81; α_pros_=0.87).

*Perceived harms of tobacco smoking* were assessed using three items from previous research: ‘Smoking can harm your health’ rated from 1=strongly disagree to 5=strongly agree, ‘How dangerous do you think it is to smoke more than 10 cigarettes a day?’ and ‘How dangerous do you think it is to smoke one or two cigarettes occasionally?’ both rated on five-point scales ranging from 1=not very dangerous to 5=very dangerous.^26^ Scale reliability in the current sample was modest (α=0.50); therefore; we analysed the items separately as in previous studies.^26–28^

*Prevalence estimates of tobacco smoking* were given on an open-ended question: ‘How many young people your age out of 100 do you think smoke tobacco cigarettes?’.^29^

*Susceptibility to tobacco smoking* was assessed using three items: ‘Do you think you will be smoking tobacco cigarettes when you are 18 years old?’; ‘Do you think you will smoke a tobacco cigarette at any time during the next year?’ and ‘If one of your friends offered you a tobacco cigarette, would you smoke it?’.^21^ ^30^ Participants who did not respond ‘definitely not’ to all three items were categorised as susceptible.

#### E-cigarette-related measures

*Appeal of using e-cigarettes* was assessed using the same three bipolar items used to assess the appeal of smoking tobacco cigarettes (α=0.88).

*Prevalence estimates of e-cigarette use* were assessed by adapting the item used to assess prevalence estimates of tobacco smoking: ‘How many young people your age out of 100 do you think use e-cigarettes?’.

#### Measures assessed only in the two conditions in which adverts were shown

*Appeal of e-cigarette adverts* was assessed by asking: ‘How much do you like this advert (not the product)?’.^31^ Responses ranged from 1=not at all to 4=a lot. Responses to the adverts were averaged into a single index (α=0.88).

*Interest in buying and trying e-cigarettes* was assessed with one item: ‘Does this advert make you want to buy and try this product?’ with scores ranging from 1=not at all to 4=yes, a lot.^31^ Responses were averaged across the 10 adverts (α=0.91).

#### Other measures for all conditions

*Tobacco smoking* was assessed using the questions ‘Have you ever smoked a tobacco cigarette?’ and ‘Have you ever tried tobacco cigarette smoking, even one or two puffs?’.^30^ Items assessing tobacco cigarette smoking were adapted to assess *use of e-cigarettes*: ‘Have you ever used an e-cigarette?’ and ‘In the past 30 days, on how many days did you use an e-cigarette?’. Gender, age, ethnicity and geographic location within the UK were also recorded.

### Procedure

ICM Direct recruited participants and collected the data. Trained interviewers knocked on doors at households from eligible LSOAs and obtained written consent from parents/guardians. Children were randomly assigned to one of the three groups, using a pre-established random sequence generated by the statistical package R. After children verbally assented, interviewers instructed them and handed over the study booklets. Interviewers assisted participants when requested. Those allocated to the glamour and healthful conditions were shown a block of 10 print-adverts in the booklets, whereas those allocated to the control condition were not shown any adverts. Children across all conditions were told that the study was about their views on e-cigarettes, and their thoughts about the e-cigarette adverts shown (only in glamour and healthful conditions) (see booklets in online supplementary materials). Exposure to the adverts was not timed, and their order was not counterbalanced. Each advert was shown only once. Children in all three groups completed the measures in one session (£10 compensation).

### Analyses

Responses to the primary and secondary outcomes were not normally distributed, so differences between the groups were assessed using non-parametric tests (Kruskal-Wallis for comparisons between more than two groups; Mann-Whitney for comparisons between two groups). Logistic regression was used for measures with dichotomous outcomes.

## Results

### Sample characteristics

Of the 564 children randomised, 153 (27.1%) were excluded because they had already smoked tobacco cigarettes or used e-cigarettes, leaving a final sample of 411 children. Sample characteristics are summarised in table 1. Randomisation was effective: the three experimental groups did not differ on any of the demographic characteristics.

**Table 1:** Demographic and smoking-related characteristics of (a) all randomised participants (n=564) and (b) participants who had never smoked tobacco cigarettes or used e-cigarettes (n=411)

(a) All randomised participants (n=564)				
	Control n=187	Glamourous adverts n=186	Health adverts n=191	Total n=564
Age, M (SD)	13.43 (1.81)	13.38 (1.64)	13.38 (1.74)	13.40 (1.73)
Gender, Male % (n)	44.4 (83)	52.2 (97)	52.4 (100)	49.6 (280)
Ethnicity, White % (n)	77.5 (145)	77.4 (144)	80.1 (153)	78.4 (442)
Ethnicity, Asian % (n)	11.8 (22)	12.4 (23)	8.4 (16)	10.8 (61)
Ethnicity, Black % (n)	5.9 (11)	4.3 (8)	5.2 (10)	5.1 (29)
Ethnicity, mixed % (n)	1.6 (3)	5.9 (11)	5.8 (11)	4.4 (25)
Ethnicity, other % (n)	3.2 (6)	0 (0)	0.5 (1)	1.2 (7)
Cigarette use, yes % (n)	22.5 (42)	20.4 (38)	20.9 (40)	21.3 (120)
Cigarette experimentation, yes % (n)	24.1 (45)	25.3 (47)	22 (42)	23.8 (134)
E-cigarette awareness, yes % (n)	88.2 (165)	88.2 (164)	84.8 (162)	87.1 (491)
E-cigarette use, yes % (n)	17.1 (32)	12.9 (24)	13.1 (25)	14.4 (81)
**(b) Final sample of non-smokers and non-users of e-cigarettes (n=411)**				

	Control n=133	Glamourous adverts n=136	Health adverts n=142	Total n=411
Age, M (SD)	13.09 (1.74)	13.14 (1.62)	13.03 (1.69)	13.09 (1.68)
Gender, Male % (n)	40.6 (54)	49.3 (67)	51.4 (73)	47.2 (19)
Ethnicity, White % (n)	72.2 (96)	72.8 (99)	76.8 (109)	74.0 (304)

### Main analyses

Descriptive statistics (M, SD) for the outcome variables are summarised in table 2. The mean ranks from non-parametric analyses are shown in online supplementary materials.

**Table 2:** Descriptive statistics (mean (SD)) of outcome measures in the three experimental groups (excluding children who had ever smoked tobacco or used e-cigarettes)

	Control n=133	Glamour n=136	Health n=142	Total n=411
Measures assessed across the three experimental conditions				
Appeal of tobacco smoking	1.21 (0.44)	1.16 (0.34)	1.20 (0.57)	1.19 (0.46)
Perceived pros of tobacco	1.81 (0.75)	1.85 (0.72)	1.92 (0.82)	1.86 (0.76)
Perceived cons of tobacco smoking	4.63 (0.45)	4.56 (0.80)	4.38 (0.98)	4.52 (0.78)
Smoking can harm your health	4.83 (0.56)	4.79 (0.77)	4.72 (0.86)	4.78 (0.74)
How dangerous is smoking more than 10 cigarettes a day?	4.68 (0.63)	4.66 (0.69)	4.61 (0.85)	4.65 (0.73)
How dangerous is smoking one or two cigarettes occasionally?	3.57 (1.03)^a,b^	3.24 (1.18)^a^	3.11 (1.28)^b^	3.30 (1.18)
Tobacco smoking prevalence estimates	32.55 (23.30)	35.19 (26.44)	29.47 (24.29)	32.37 (24.78)
Appeal of using e-cigarettes	1.58 (0.80)	1.66 (0.86)	1.65 (0.80)	1.63 (0.82)
E-cigarette use prevalence estimates	17.45 (15.55)^a^	25.06 (24.27)^a,b^	18.16 (20.42)^b^	20.24 (21.20)
Measures assessed only in the two conditions were adverts were shown				
Appeal of e-cigarette adverts	–	1.74 (0.63)	1.83 (0.63)	1.79 (0.63)
Interest in buying and trying e-cigarettes	–	1.36 (0.49)	1.44 (0.57)	1.40 (0.53)

Means (SDs) in the same row with same letters are significantly different at p<0.05.

#### Tobacco-related outcomes

There were no statistically significant differences between the groups on appeal of smoking tobacco cigarettes, perceived pros and cons of smoking tobacco cigarettes, susceptibility to smoking tobacco cigarettes or the prevalence estimates for tobacco smoking. Of the three items assessing the perceived harms of smoking tobacco cigarettes, there was a difference between the groups on the item, ‘How dangerous do you think it is to smoke one or two cigarettes occasionally?’. Children exposed to either set of e-cigarette adverts perceived the danger as lower than the control group (Kruskall-Wallis test, χ^2^(2)=10.07, p=0.007). Those in the glamour (U=7680.500, Z=−2.225, p=0.026, r=0.136) and those in the health condition (U=7492.000, Z=−3.057, p=0.002, r=0.184) perceived occasional smoking of one or two tobacco cigarettes to be less harmful than did those in the control condition. There was no significant difference in perceived harm of occasional smoking between participants in the glamour and health conditions (U=9054.000, Z=−0.926, p=0.354, r=0.045; see online supplementary materials table S2).

#### E-cigarette-related outcomes

The appeal of using e-cigarettes did not differ between experimental groups, but estimates of the prevalence of e-cigarette use differed significantly between conditions (Kruskal-Wallis test, χ^2^(2)=6.95, p=0.031), with those in the glamour condition estimating that more children were using e-cigarettes compared with the control group (U=7461.000, Z=−2.213, p=0.027, r=0.136) and the health group (U=7981.500, Z=−2.334, p=0.020, r=0.140). There was no significant difference in prevalence estimates of e-cigarette use between children in the health and control groups (U=9003.000, Z=−0.153, p=0.879, r=0.009).

#### Outcomes assessed only in the groups exposed to e-cigarette adverts

Children exposed to either set of adverts did not differ in how appealing they found the adverts or their interest in buying or trying e-cigarettes, both of which were low.

### Exploratory analyses

**Figure 1:**
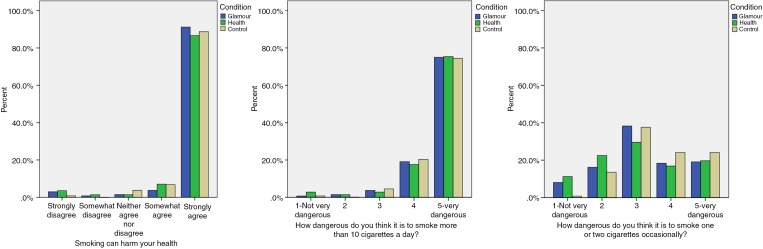
Proportions (%) of participants responding to each response option for each of the three items measuring perceived harm of smoking tobacco cigarettes.

Two sets of post hoc analyses were conducted to explore the finding that exposure to either set of e-cigarette adverts reduced the perceived harm of occasional smoking of tobacco cigarettes. First, we examined participants' responses to the three harm items across the three experimental conditions (see figure 1 and online supplementary materials, table S3). We found that exposure to either set of adverts increased the number of participants who perceived occasional smoking of one or two tobacco cigarettes as ‘not very dangerous’.

We used the same measures of perceived harm of tobacco smoking in another study assessing the impact of exposure to candy-like flavoured and non-flavoured e-cigarette adverts.^9^ Using results from this study and the current study, we conducted a meta-analysis of the proportions of participants who responded to the item, ‘How dangerous do you think it is to smoke one or two cigarettes occasionally?’ by stating that it was ‘not very dangerous’, comparing those exposed to any type of advert for e-cigarettes with those in the control groups.

**Figure 2:**
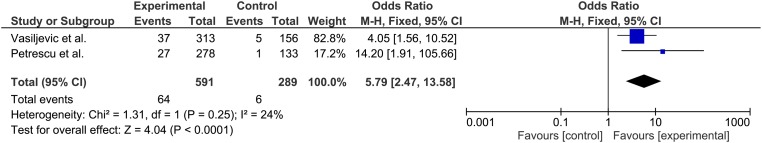
Forest plot of meta-analysis of impact of exposure to e-cigarette adverts on the perception that occasional smoking of one or two cigarettes is not very dangerous.

Children's exposure to any of the adverts for e-cigarettes used in each of the two studies increased the perception that occasional smoking of one or two cigarettes was not very dangerous: OR=5.79, 95% CI (2.47 to 13.58), I^2^=24%, Z=4.04, p<0.001 (see figure 2).

## Discussion

In an experiment examining the effects of glamorous and healthful e-cigarette adverts among UK 11–16 year olds, exposure to the adverts had no impact on the appeal, susceptibility or perceived benefits of smoking tobacco cigarettes. While the perceived harm of smoking more than 10 cigarettes per day was similar across groups, those exposed to either set of adverts perceived the harms of smoking one or two tobacco cigarettes occasionally to be lower than did those in the control group. The lack of impact of the adverts on appeal and susceptibility to tobacco smoking is encouraging and replicates findings from two other studies using similar measures.^8^ ^9^ However, the impact on perceived harms is concerning given that harm perceptions are predictive of tobacco smoking.^32–34^ Perceived harm of occasional smoking is particularly predictive of subsequent engagement with smoking^35^ ^36^ and differentiates smokers from non-smokers.^37^ Furthermore, although the health consequences of occasional smoking can be as severe as regular smoking,^38^ young smokers who smoke occasionally do not consider themselves smokers, believing that they are immune to the risks associated with smoking, with low intentions to quit.^39^ ^40^ Although the adverts did not affect perceptions of appeal, susceptibility and perceived benefits of smoking tobacco cigarettes, their effect on perceived harm is theoretically and empirically important. In theories like the Health Belief Model, perceived threat is a key construct affecting changes in health behaviour. In the empirical literature, perceived risk significantly predicts intentions and behaviours generally,^41^ ^42^ as well as more specifically in relation to smoking.^32–34^

Only two other studies thus far have examined the perceived harms of tobacco smoking following exposure to e-cigarette adverts. Vasiljevic and colleagues found no significant differences in perceived harm of regular and occasional tobacco smoking following exposure to either candy-like flavoured or non-flavoured e-cigarette adverts among English school children.^9^ However, meta-analysis using data from that study and the current study showed that exposure to any kind of e-cigarette advert reduced the perceived harm of occasional smoking one or two tobacco cigarettes. In contrast, Farrelly and colleagues' experiment found that exposing children to four e-cigarette TV adverts did not decrease the perceived harm of tobacco smoking.^8^ However, their measure of perceived harm (‘How harmful are cigarettes’) was general and not time-specific. A similar item (‘Smoking can harm your health’) that was used in the present study and the study by Vasiljevic *et al*^9^ replicated the results by Farrelly *et al*^8^ in showing that brief exposure to e-cigarette adverts did not affect children's general perceptions that smoking is harmful. A broader set of items assessing harm, potentially specifying type of harm, time frame and under what conditions the harm would occur should be used in future studies alongside qualitative studies exploring how children perceive harms of tobacco smoking when exposed to e-cigarette adverts.^43^

The present study also found that children exposed to glamourous e-cigarette adverts estimated the number of young e-cigarette users to be greater than did children exposed either to healthful e-cigarette adverts or no adverts. This extends recent findings that exposure to e-cigarette adverts fosters more positive attitudes and intentions to use e-cigarettes in children, suggesting that exposure to e-cigarette adverts may shift the perceived norms of e-cigarette use among children.^8^ ^9^ This may contribute to the increasing prevalence of e-cigarette use among children. We only observed this effect for glamorous depictions of e-cigarette use. That we did not find a similar effect for healthful adverts suggests that glamorous depictions may be more potent at shifting the norms of e-cigarette use among children. Our findings suggest that restricting youth-targeted advertising with glamorous images of e-cigarettes may curb the rising experimentation and use of e-cigarettes in young people, but this requires evaluation.

### Strengths and limitations

The current study provides novel, robust and timely evidence contributing to the small but growing evidence base on the potential for e-cigarettes to influence attitudes towards smoking tobacco cigarettes. The study is limited by assessing perceptions and attitudes and not actual tobacco smoking or e-cigarette use. However, there is a large body of evidence demonstrating that perceptions and attitudes influence many judgements and behaviours.^44^ ^45^ In keeping with this, the appeal of tobacco smoking predicts subsequent tobacco smoking in young people.^29^ ^46^ Nevertheless, future studies should examine more direct measures of behaviour or incorporate implicit measures of appeal that avoid social desirability biases.

The study was also limited by using momentary exposure to still e-cigarette adverts. The reported effects may therefore underestimate the impact of the prolonged and vivid exposure to e-cigarette adverts that children experience in real-life settings (on television, internet and social media). The present study should be extended to examine children's responses to e-cigarette adverts in more naturalistic settings, over longer periods of time and using more vivid forms of advertisement. The advertising stimuli depicted primarily ‘cigalike’ devices, rather than advanced generation devices, which could limit generalizability across the range of e-cigarettes currently available in the marketplace. However, the choice to focus on first-generation devices was deliberate, given that we wished to explore how devices that looked like tobacco cigarettes impacted beliefs about or interest in tobacco cigarettes. Future studies could explore differential effects of first-generation and second-generation e-cigarettes.

### Implications for policy

Since May 2016, e-cigarette marketing across Europe falls under the new Tobacco Products Directive (TPD).^47^ The new regulations limit the exposure of children to TV and newspaper e-cigarette adverts. However, the proposed implementation of these regulations in the UK and other EU member states still allows some form of e-cigarette advertising (posters, leaflets, billboards in shops), so children may still be exposed to e-cigarette adverts. The TPD also does not explicitly prohibit the use of glamorous or healthful themes/content. In the USA, the Food and Drug Administration recently began regulating e-cigarettes, but the regulations do not include provisions to limit youth exposure to e-cigarette advertising or to restrict e-cigarette adverts with potentially youth-appealing themes/content.^48^

From a policy viewpoint, we note that our study only examined in-the-moment responses to e-cigarette adverts, and therefore our findings may underestimate or overestimate the impact these adverts may have in the longer term. Contemporary marketing communications are aimed not so much at stimulating immediate purchasing,^49^ but doing so more indirectly by raising awareness, interest and identification with products and brands.^50^ ^51^ These outcomes are subtle and develop gradually and are unlikely to be observed in studies such as ours investigating the immediate effects of advertising.

However, this is the first study to provide evidence for the possible cross-cueing and re-normalising effects of e-cigarettes on tobacco smoking^52^ ^53^ by showing that associating e-cigarettes with either glamour or health lowers the perceived harm of occasional smoking of one or two tobacco cigarettes among children who have not used tobacco cigarettes or e-cigarettes. Moreover, our study shows that glamorous e-cigarette adverts can shift the perceived normativeness of e-cigarette use by increasing the perceived prevalence of children who are e-cigarette users. These findings, coupled with the growing popularity of e-cigarette products among children,^2^ ^54^ ^55^ and the wider literature on the dangers to the developing brain arising from nicotine exposure and addiction^16^ ^56^ suggest a need to re-examine the rules surrounding the marketing of e-cigarettes.

**What this paper adds**

- Exposure of adolescents to e-cigarette adverts increases the appeal of using e-cigarettes.
- Given the similarities in appearance between e-cigarettes and tobacco cigarettes, could exposure to e-cigarette adverts increase the appeal of smoking tobacco cigarettes?
- Exposing children to e-cigarette adverts associating e-cigarettes with glamour or their putative health benefits did not increase the appeal or perceived benefits of smoking tobacco cigarettes.
- Exposing children to either set of e-cigarette adverts did, however, lower their perceptions of the harm of smoking one or two tobacco cigarettes occasionally.
- This study provides the first evidence that exposure to e-cigarette adverts might influence children's perceptions of smoking tobacco cigarettes, reducing their perceived harm.
