# Supplementary material for: What is the impact of e-cigarette adverts on children's perceptions of tobacco smoking? An experimental study
Source: Tob Control. 2016 Sep 5;26(4):421–7. doi: 10.1136/tobaccocontrol-2016-052940 (PMC5520264; doi:10.1136/tobaccocontrol-2016-052940)
Supplement: supplementary materials [file tobaccocontrol-2016-052940supp001.pdf]

ONLINE SUPPLEMENTARY MATERIALS:

WHAT IS THE IMPACT OF E-CIGARETTE ADVERTS ON CHILDREN'S PERCEPTIONS OF TOBACCO SMOKING? AN EXPERIMENTAL STUDY

Petrescu, D. C., Vasiljevic, M., Pepper, J. K., Ribisl, K. M., & Marteau, T. M.

Pilot Study	pp. 2-7
Main Study (Supplementary Tables)	pp. 8-10
Glamour Group Booklet	pp. 11-33
Health Group Booklet	pp. 34-56
Control Group Booklet	pp. 57-68

Pilot Study

Methods

Design

The study consisted of an online survey in which participants rated e-cigarette adverts with regard to how "cool" and "healthy" they perceived them to be.

Participants and recruitment

Participants aged 16 (n = 255) took part in the pilot study. Participants were recruited by Onepoll [http://www.onepoll.com/], a market research agency, who sent invitations to members of their panel to allow their appropriately aged children take part in the study. Electronic consent was obtained from both children and parents.

Procedure

Pictures were selected from a large number of e-cigarette adverts sourced online. Two authors DCP and MV rated the adverts with regard to how healthy and glamourous their content was. A total of 40 adverts were selected to be piloted. In order to minimise fatigue, participants were divided into two groups rating 20 adverts each. Participants rated each advert on two 7-point scales ranging from *Uncool (1)* to *Cool (7)* and from *Unhealthy (1)* to *Healthy (7)*.

Data analysis

Of the initial 255 participants, 119 (46.7%) had experimented with tobacco cigarettes in the past and were excluded from analyses. The final analysis was based on 136 participants who had never smoked.

Ratings on the two bipolar items were not normally distributed, therefore data were analysed using non-parametric tests. For each of the 40 adverts, Wilcoxon signed-rank tests were conducted to compare their “health” and “cool” ratings.

Results

Ten adverts were selected to be shown to participants in the glamour condition of the main study. All 10 adverts were selected on the basis of showing significantly higher scores on “cool” ratings as compared to “health” ratings as indicated by Wilcoxon signed-rank tests. Only 6 adverts showed significantly higher ranks for “health” ratings as compared to “cool” ratings and were selected to be used in the main study. One advert (Advert 40) showed a marginally significant difference in the Wilcoxon signed-rank test ($Z = -1.800$, $p = .072$, $r = -.22$) and was also included in the main study. Three additional adverts were selected to be shown in the health condition on the basis of having high health scores albeit these were not significantly higher than their “cool” ratings. Results from each of the 40 Wilcoxon signed-rank tests are summarised in Table 1 below. A visual representation of all scores is presented in terms of means, for ease of intrepretation (see Figure 1).

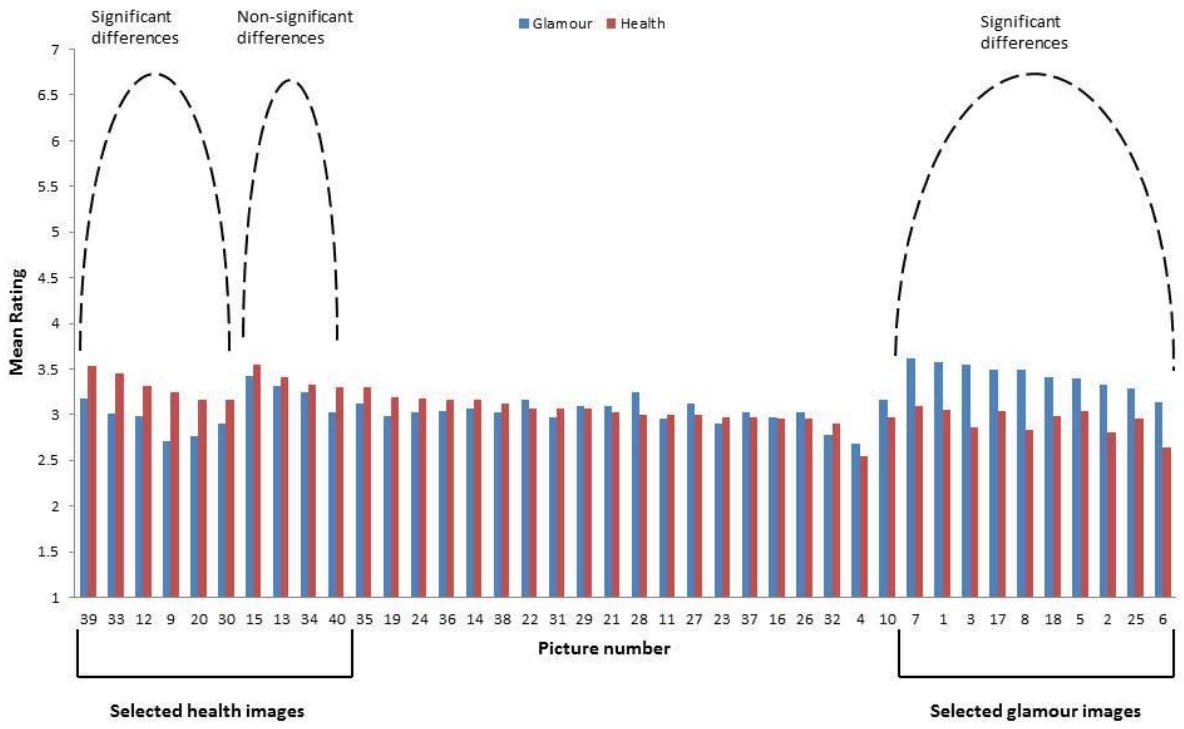

Figure 1. Pilot study results. Mean ratings for each of the 40 adverts tested are shown (10 health and 10 glamour adverts were selected to be used in the main study).

To ensure that the 20 adverts identified for the main study successfully discriminate between glamour and health, we performed a by-item analysis. We collapsed participants' ratings for each advert to create average ratings, one on the glamour dimension and one on the health dimension. The resulting data were normally distributed, and we thus proceeded with between-item *t*-tests to compare the health ratings for adverts allocated to the health group (*M* = 3.34, *SD* = .14) with the health ratings for adverts allocated to the glamour group (*M* = 2.93, *SD* = .14), which were significantly different, $t(18) = -6.36$, $p < .001$, d = 2.99. Likewise, we also compared the glamour ratings for adverts allocated to the glamour group (*M* = 3.43, *SD* = .15) with the glamour ratings for adverts allocated to the health group (*M* =

3.06, *SD* = .23), which were again significantly different, $t(18) = 4.20$, $p = .001$, d = 1.98. Additional *t*-tests confirmed that adverts allocated to the health group had higher health ratings than glamour ratings, $t(18) = -3.29$, $p = .004$, d = 1.55, and adverts allocated to the glamour group had higher glamour ratings than health ratings, $t(18) = 7.49$, $p < .001$, d = 3.53. Please see Figure 2 for a graphical representation of these results.

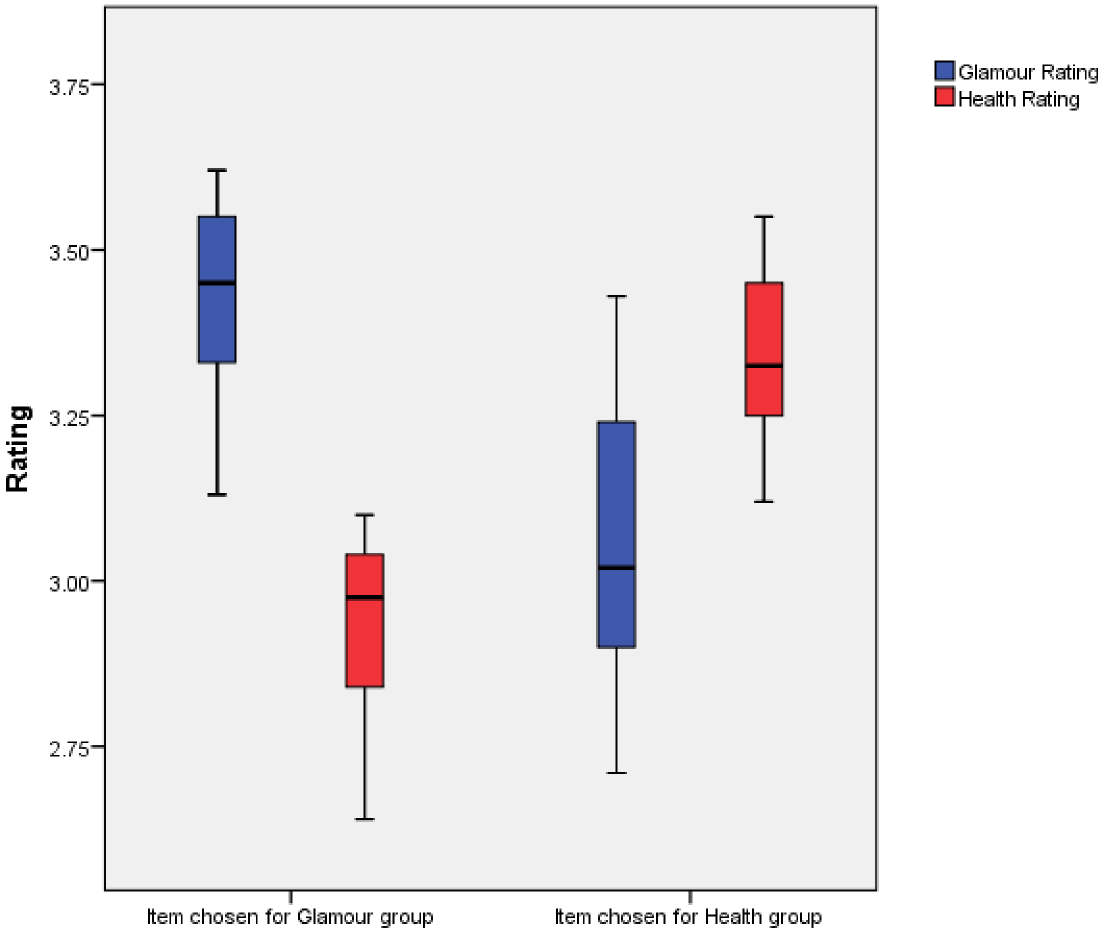

Figure 2. Boxplot illustrating the allocation of adverts into a Glamour group (10 adverts) and a Health group (10 adverts) based on health and glamour ratings obtained in the pilot study.

Table 1. Median scores for "Cool" and "Health" ratings and Wilcoxon signed difference ranks for each of the 40 adverts

	**Cool ratings** *Mdn*	**Health ratings** *Mdn*	**Negative ranks** *Mean rank (n)*	**Positive ranks** *Mean rank (n)*	**Ties** *n*	**Effect size** ***r***	**Selected** *Yes/No*
Picture 1 (n = 69)	4.00[a]	3.00[a]	11.50 (10)	20.60 (25)	34	-.41	Yes
Picture 2 (n = 69)	4.00[a]	3.00[a]	12.00 (8)	19.78 (27)	34	-.45	Yes
Picture 3 (n = 69)	4.00[a]	3.00[a]	12.50 (8)	21.37 (30)	31	-.49	Yes
Picture 4 (n = 69)	2.00[a]	2.00[b]	12.50 (11)	15.03 (16)	42	-.16	No
Picture 5 (n = 69)	4.00[a]	3.00[a]	11.00 (10)	18.38 (21)	38	-.34	Yes
Picture 6 (n = 69)	3.00[a]	2.00[a]	12.54 (14)	22.30 (22)	33	-.31	Yes
Picture 7 (n = 69)	4.00[a]	3.00[a]	15.13 (8)	18.85 (27)	34	-.39	Yes
Picture 8 (n = 69)	4.00[a]	3.00[a]	13.33 (9)	20.82 (28)	32	-.43	Yes
Picture 9 (n = 69)	2.00[a]	4.00[a]	16.88 (24)	10.00 (6)	39	-.44	Yes
Picture 10 (n = 69)	3.00[a]	3.00[b]	15.67 (12)	17.76 (21)	36	-.21	No
Picture 11 (n = 69)	3.00[a]	3.00[b]	13.60 (15)	14.50 (12)	42	-.05	No
Picture 12 (n = 69)	3.00[a]	4.00[a]	16.38 (20)	11.94 (9)	40	-.30	Yes
Picture 13 (n = 69)	4.00[a]	4.00[b]	10.88 (13)	11.19 (8)	48	-.11	Yes
Picture 14 (n = 69)	3.00[a]	3.00[b]	16.97 (15)	14.03 (15)	39	-.06	No
Picture 15 (n = 69)	4.00[a]	4.00[b]	18.42 (18)	15.30 (15)	36	-.12	Yes
Picture 16 (n = 69)	3.00[a]	3.00[b]	14.63 (16)	16.50 (14)	39	-.00	No
Picture 17 (n = 69)	4.00[a]	3.00[a]	15.81 (13)	22.10 (26)	30	-.32	Yes
Picture 18 (n = 69)	4.00[a]	3.00[a]	10.00 (7)	16.59 (22)	40	-.40	Yes
Picture 19 (n = 69)	3.00[a]	3.00[b]	17.05(21)	16.92 (12)	36	-.18	No
Picture 20 (n = 69)	3.00[a]	3.00[a]	17.32 (22)	10.50(8)	35	-.38	Yes
Picture 21 (n = 67)	3.00[a]	3.00[b]	17.44 (16)	18.47 (19)	32	-.08	No

Picture 22 (n = 67)	4.00[a]	3.00[b]	18.67 (12)	15.20 (20)	35	-.10	No
Picture 23 (n = 67)	3.00[a]	3.00[b]	14.58 (18)	16.88 (12)	37	-.08	No
Picture 24 (n = 67)	3.00[a]	4.00[a]	14.58 (20)	17.35 (10)	37	-.16	No
Picture 25 (n = 67)	4.00[a]	3.00[b]	12.96 (12)	18.63 (20)	35	-.26	Yes
Picture 26 (n = 67)	3.00[a]	3.00[b]	14.21 (12)	14.72 (16)	39	-.09	No
Picture 27 (n = 67)	3.00[a]	3.00[b]	18.30 (15)	17.78 (20)	32	-.08	No
Picture 28 (n = 69)	4.00[a]	3.00[b]	16.09 (16)	20.43 (20)	31	-.15	No
Picture 29 (n = 67)	3.00[a]	3.00[b]	14.83 (12)	13.33 (15)	40	-.03	No
Picture 30 (n = 67)	3.00[a]	3.00[a]	12.53 (19)	14.50 (6)	42	-.26	Yes
Picture 31 (n = 67)	3.00[a]	3.00[b]	12.73 (13)	11.05 (10)	44	-.11	No
Picture 32 (n = 67)	2.00[a]	3.00[b]	18.26 (17)	14.50 (15)	35	-.11	No
Picture 33 (n = 67)	3.00[a]	4.00[a]	16.34 (22)	10.79 (7)	38	-.39	Yes
Picture 34 (n = 67)	3.00[a]	4.00[b]	17.88 (16)	14.00 (15)	36	-.10	No
Picture 35 (n = 67)	3.00[a]	3.00[b]	14.57 (14)	11.00 (11)	42	-.14	Yes
Picture 36 (n = 67)	3.00[a]	3.00[b]	16.61 (18)	15.15 (13)	36	-.13	No
Picture 37 (n = 67)	3.00[a]	3.00[b]	12.86 (14)	15.23 (13)	40	-.03	No
Picture 38 (n = 67)	3.00[a]	3.00[b]	15.66 (19)	17.73 (13)	35	-.08	No
Picture 39 (n = 67)	3.00[a]	4.00[a]	16.38 (21)	13.44 (9)	37	-.30	Yes
Picture 40 (n = 67)	3.00[a]	4.00[b]	14.38 (17)	11.83 (9)	41	-.22	Yes

Note: Medians in the same row with same letters are significantly different at $p<.05$

Main study

Table 2. Non-parametric mean ranks of outcome measures in the three experimental groups (excluding children who had ever smoked tobacco or used e-cigarettes)

	**Control**<br>Mean rank<br>*n* = 133	**Glamour**<br>Mean rank<br>*n* = 136	**Health**<br>Mean rank<br>*n* = 142
**Measures assessed across the three experimental conditions (Kruskal-Wallis)**			
Appeal of tobacco smoking	212.47	207.64	198.37
Perceived pros of tobacco smoking	197.67	207.54	212.32
Perceived cons of tobacco smoking	208.29	215.37	194.89
Smoking can harm your health?	206.23	210.58	201.40
How dangerous is smoking more than 10 cigarettes a day?	205.61	206.27	206.11
How dangerous is smoking one or two cigarettes occasionally?	230.92[a, b]	200.40[a]	188.02[b]
Tobacco smoking prevalence estimates	206.28	215.18	189.58
Appeal of using e-cigarettes	196.58	207.21	213.66
E-cigarette use prevalence estimates	193.64[a]	224.95[a, b]	191.82[b]

Table 2 continued

**Susceptibility to smoking tobacco – dichotomous outcome (Logistic Regression)**			
Susceptibility	*n (%)*	*n (%)*	*n (%)*
	63 (33.7%)	79 (42.5%)	72 (37.7%)
**Measures assessed only in the two conditions were adverts were shown (Mann-Whitney)**			
		Mean rank	Mean rank
Appeal of e-cigarette adverts	/	133.26	145.28
Interest in buying and trying e-cigarettes	/	133.45	145.29

Note: Mean ranks in the same row with same letters are significantly different at p<.05

Table 3. Frequencies and percentages of participants' response ratings on the question:

"How dangerous do you think it is to smoke one or two cigarettes occasionally?"

	**Control** *n* = 187	**Glamourous Adverts** *n* = 186	**Health Adverts** *n* = 191
Not very dangerous - % (n)	0.8 (1)	8.1 (11)	11.3 (16)
Not dangerous - % (n)	13.5 (18)	16.2 (22)	22.5 (32)
Neither - % (n)	37.6 (50)	38.2 (52)	29.6 (42)
Dangerous - % (n)	24.1 (32)	18.4 (25)	16.9 (24)
Very dangerous - % (n)	24.1 (32)	19.1 (26)	19.7 (28)

"Study on e-cigarettes"

- Please fill in this form carefully.
- There are no right or wrong answers. We are interested only in what you think.
- It is important that the information you give is accurate and truthful.
- The researchers have no way of finding out who you are so you can be totally honest (please don't write your name on this survey).

Study details

An e-cigarette is an electronic device that runs on a battery and produces vapour instead of smoke.

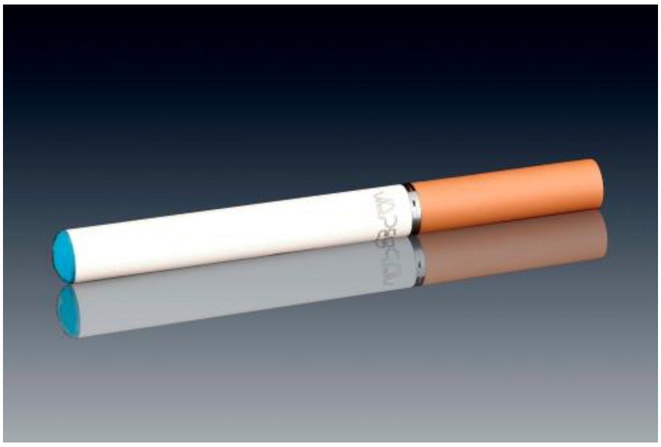

The aim of this study is to describe what people like you think about adverts for e-cigarettes.

What the study involves:

- You will be shown 10 e-cigarette adverts and asked to rate each one
- You will then be asked several questions about your thoughts on smoking tobacco cigarettes and using e-cigarettes
- You will also be asked several general questions about you
- For each question please cross the circle that best describes your views. For example, if you like the advert quite a lot you would cross the third circle as shown below:

How much do you like this advert (not the product)?

Not at all			*A lot*
1	2	3	4
○	○	⊗	○

Next, you will be shown 10 e-cigarette adverts and asked to rate each one.

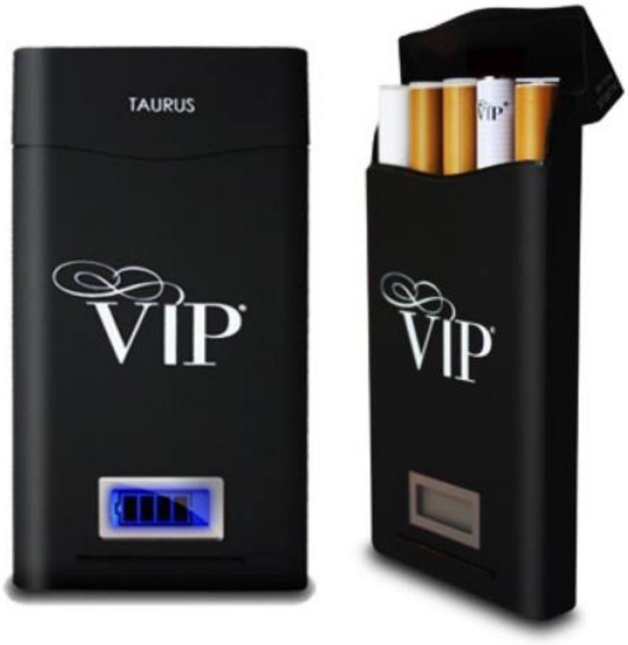

1. **How much do you like this advert (not the product)?**

Not at all			*A lot*
1	2	3	4
○	○	○	○

2. **Does this advert make you want to buy and try this product?**

Not at all			*Yes, a lot*
1	2	3	4
○	○	○	○

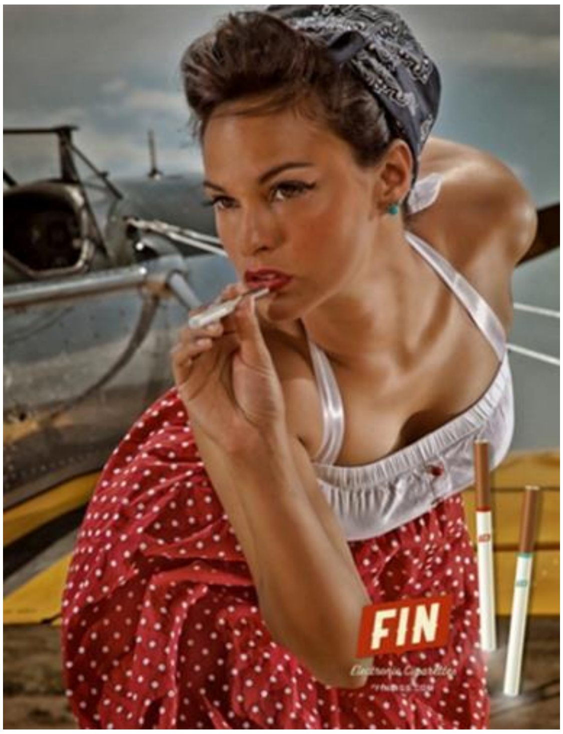

3. How much do you like this advert (not the product)?

Not at all			*A lot*
1	2	3	4
○	○	○	○

4. Does this advert make you want to buy and try this product?

Not at all			*Yes, a lot*
1	2	3	4
○	○	○	○

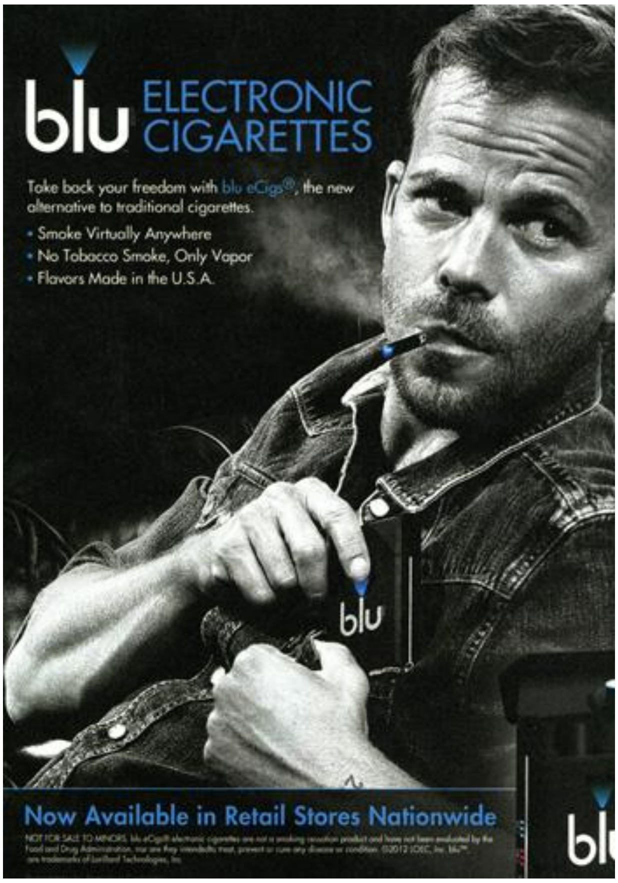

5. How much do you like this advert (not the product)?

Not at all			*A lot*
1	2	3	4
○	○	○	○

6. Does this advert make you want to buy and try this product?

Not at all			*Yes, a lot*
1	2	3	4
○	○	○	○

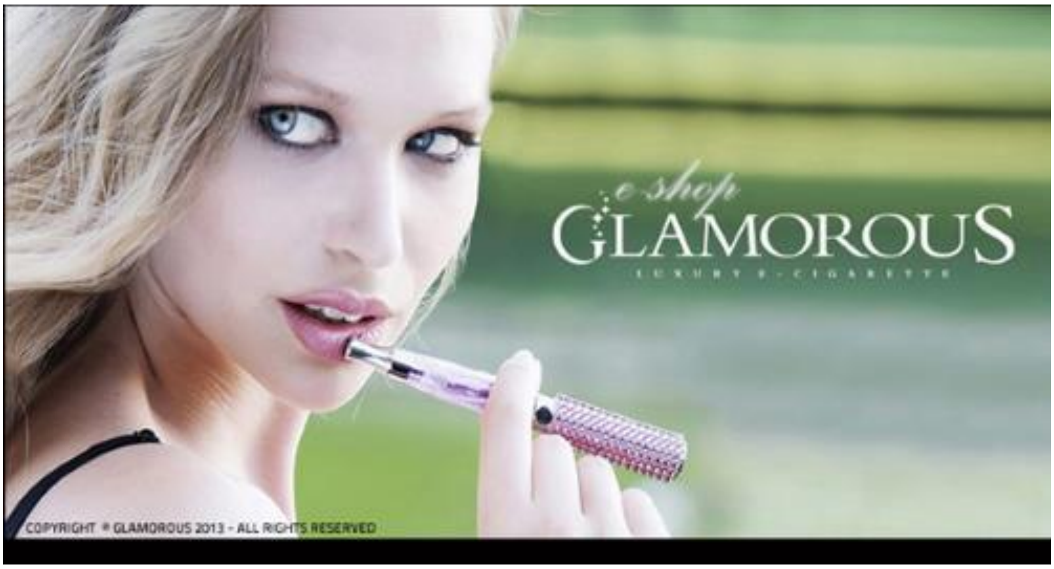

7. How much do you like this advert (not the product)?

Not at all			*A lot*
1	2	3	4
○	○	○	○

8. Does this advert make you want to buy and try this product?

Not at all			*Yes, a lot*
1	2	3	4
○	○	○	○

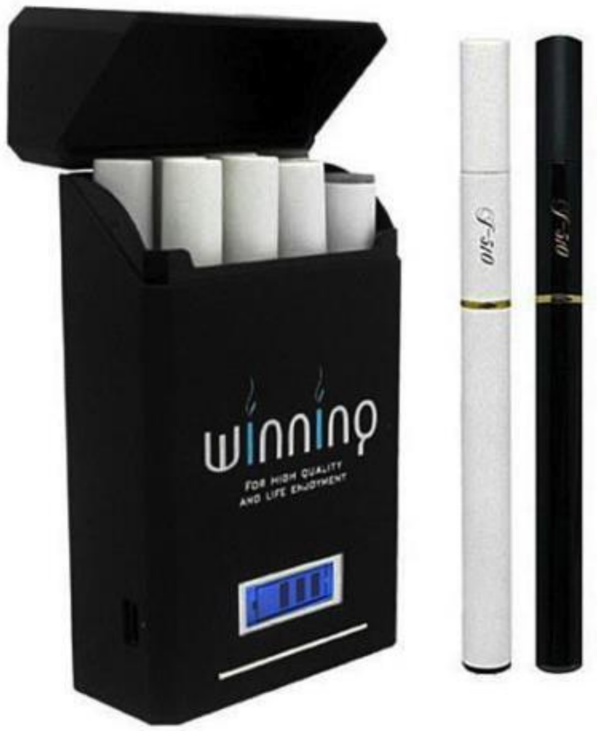

9. How much do you like this advert (not the product)?

Not at all			*A lot*
1	2	3	4
○	○	○	○

10. Does this advert make you want to buy and try this product?

Not at all			*Yes, a lot*
1	2	3	4
○	○	○	○

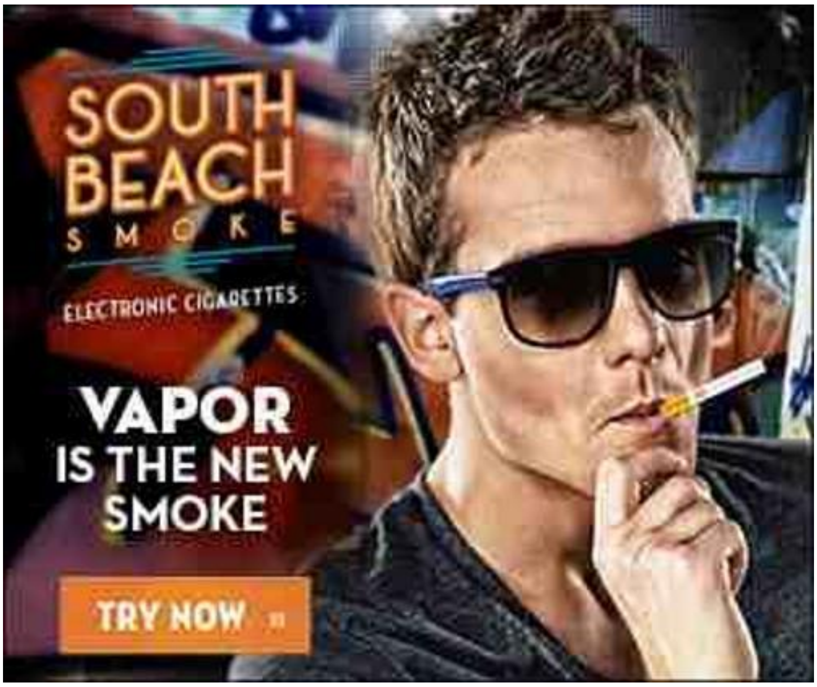

11. How much do you like this advert (not the product)?

Not at all			*A lot*
1	2	3	4
○	○	○	○

12. Does this advert make you want to buy and try this product?

Not at all			*Yes, a lot*
1	2	3	4
○	○	○	○

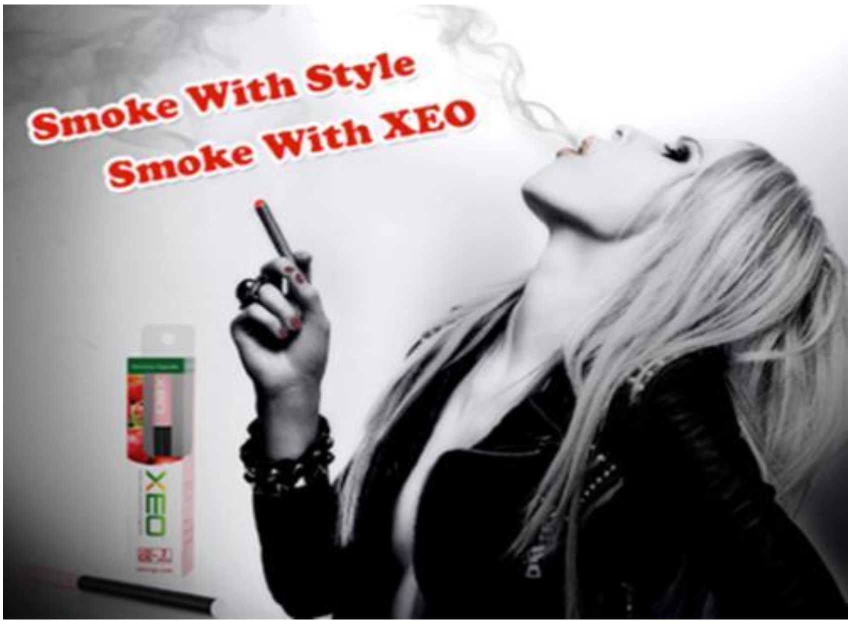

13. How much do you like this advert (not the product)?

Not at all			*A lot*
1	2	3	4
◯	◯	◯	◯

14. Does this advert make you want to buy and try this product?

Not at all			*Yes, a lot*
1	2	3	4
◯	◯	◯	◯

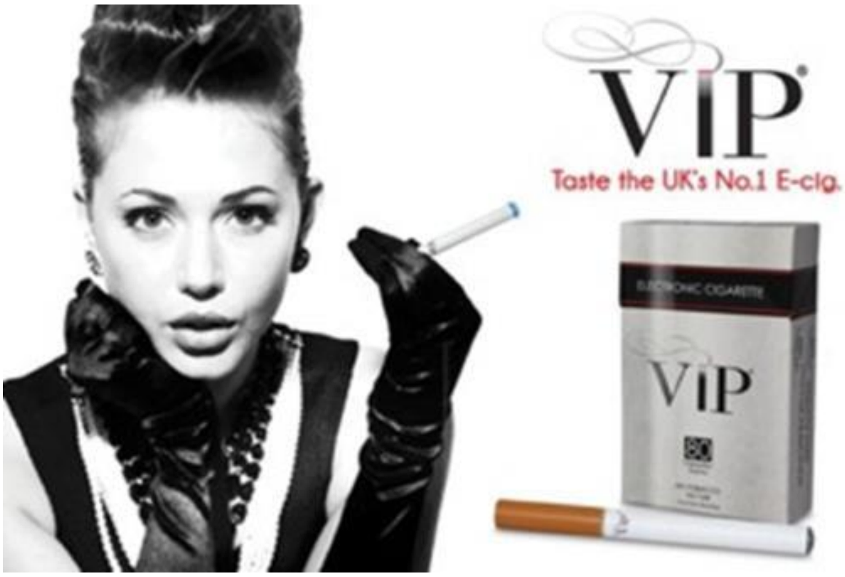

15. How much do you like this advert (not the product)?

Not at all			*A lot*
1	2	3	4
◯	◯	◯	◯

16. Does this advert make you want to buy and try this product?

Not at all			*Yes, a lot*
1	2	3	4
◯	◯	◯	◯

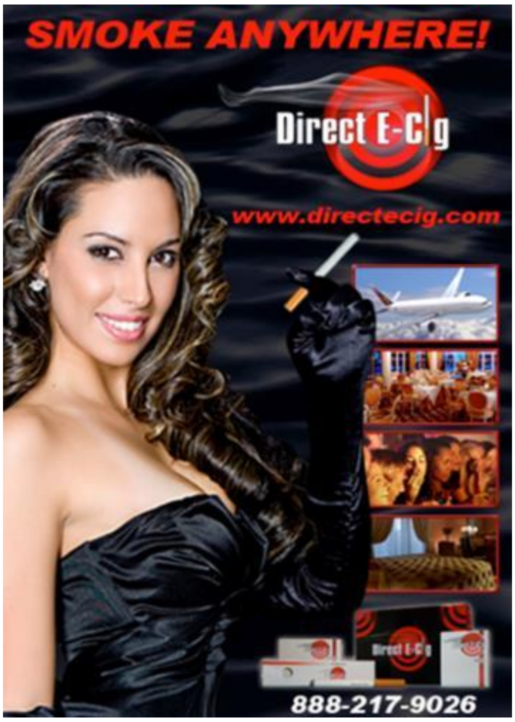

17. How much do you like this advert (not the product)?

Not at all			*A lot*
1	2	3	4
◯	◯	◯	◯

18. Does this advert make you want to buy and try this product?

Not at all			*Yes, a lot*
1	2	3	4
◯	◯	◯	◯

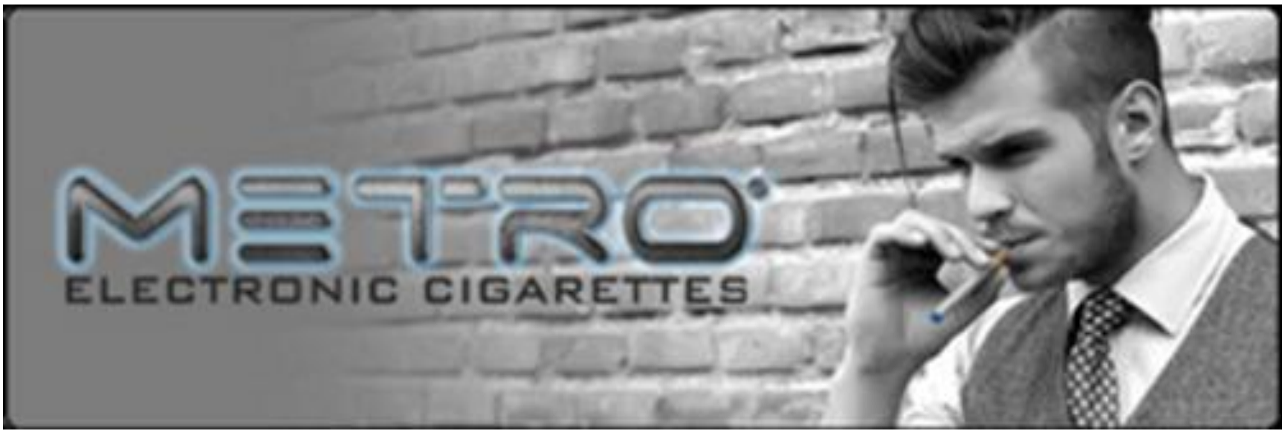

19. How much do you like this advert (not the product)?

Not at all			*A lot*
1	2	3	4
○	○	○	○

20. Does this advert make you want to buy and try this product?

Not at all			*Yes, a lot*
1	2	3	4
○	○	○	○

The next questions ask about tobacco cigarettes.

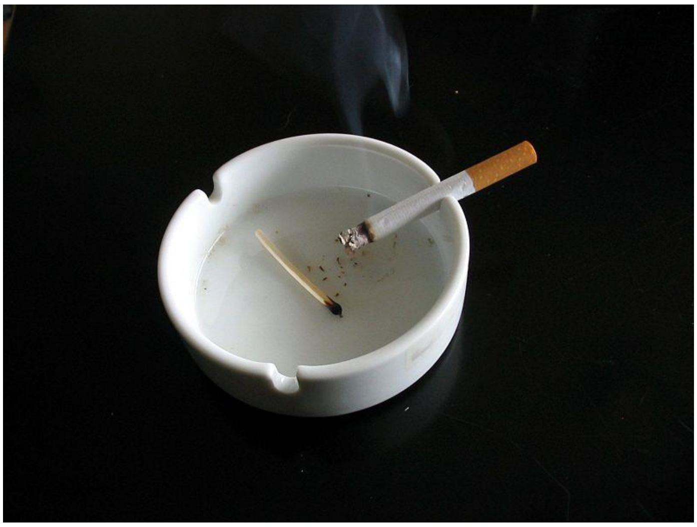

21. Please cross the circles for (a), (b) and (c) that best describe how you feel about smoking tobacco cigarettes:

a)

Unattractive				*Attractive*
1	2	3	4	5
○	○	○	○	○

b)

Not cool				*Cool*
1	2	3	4	5
○	○	○	○	○

c)

Boring				*Fun*
1	2	3	4	5
○	○	○	○	○

Please cross the circle that best describes your views on each of the next 12 statements:

22. Kids who smoke get more respect from others.

Strongly disagree	*Somewhat disagree*	*Neither agree nor disagree*	*Somewhat agree*	*Strongly agree*
1	2	3	4	5
○	○	○	○	○

23. Kids who smoke have more friends.

Strongly disagree	*Somewhat disagree*	*Neither agree nor disagree*	*Somewhat agree*	*Strongly agree*
1	2	3	4	5
○	○	○	○	○

24. Kids who smoke go out on more dates.

Strongly disagree	*Somewhat disagree*	*Neither agree nor disagree*	*Somewhat agree*	*Strongly agree*
1	2	3	4	5
○	○	○	○	○

25. Smoking helps people cope better with frustrations.

Strongly disagree	*Somewhat disagree*	*Neither agree nor disagree*	*Somewhat agree*	*Strongly agree*
1	2	3	4	5
○	○	○	○	○

26. Smoking is pleasurable.

Strongly disagree	*Somewhat disagree*	*Neither agree nor disagree*	*Somewhat agree*	*Strongly agree*
1	2	3	4	5
○	○	○	○	○

27. Smoking cigarettes relieves tension.

Strongly disagree	*Somewhat disagree*	*Neither agree nor disagree*	*Somewhat agree*	*Strongly agree*
1	2	3	4	5
○	○	○	○	○

28. Smoking stinks.

Strongly disagree	*Somewhat disagree*	*Neither agree nor disagree*	*Somewhat agree*	*Strongly agree*
1	2	3	4	5
○	○	○	○	○

29. Smoking is a messy habit.

Strongly disagree	*Somewhat disagree*	*Neither agree nor disagree*	*Somewhat agree*	*Strongly agree*
1	2	3	4	5
○	○	○	○	○

30. Smoking makes teeth yellow.

Strongly disagree	*Somewhat disagree*	*Neither agree nor disagree*	*Somewhat agree*	*Strongly agree*
1	2	3	4	5
○	○	○	○	○

31. Smoking can affect the health of others.

Strongly disagree	*Somewhat disagree*	*Neither agree nor disagree*	*Somewhat agree*	*Strongly agree*
1	2	3	4	5
○	○	○	○	○

32. Smoking cigarettes is hazardous to people's health.

Strongly disagree	*Somewhat disagree*	*Neither agree nor disagree*	*Somewhat agree*	*Strongly agree*
1	2	3	4	5
○	○	○	○	○

33. Cigarette smoke bothers other people.

Strongly disagree	*Somewhat disagree*	*Neither agree nor disagree*	*Somewhat agree*	*Strongly agree*
1	2	3	4	5
○	○	○	○	○

The following 3 questions ask whether you might smoke <u>tobacco cigarettes</u> in the future.

34. Do you think you will smoke a <u>tobacco cigarette</u> at any time during the next year?

Definitely not	*Probably not*	*Probably yes*	*Definitely yes*
1	2	3	4
○	○	○	○

35. Do you think you will be smoking <u>tobacco cigarettes</u> when you are 18 years old?

Definitely not	*Probably not*	*Probably yes*	*Definitely yes*
1	2	3	4
○	○	○	○

36. If one of your friends offered you a <u>tobacco cigarette</u>, would you smoke it?

Definitely not	*Probably not*	*Probably yes*	*Definitely yes*
1	2	3	4
○	○	○	○

In the following three questions please cross the circles that best describe your opinions on smoking tobacco cigarettes.

37. Smoking can harm your health.

Strongly disagree				*Strongly agree*
1	2	3	4	5
○	○	○	○	○

38. How dangerous do you think it is to smoke more than 10 cigarettes a day?

Not very dangerous				*Very dangerous*
1	2	3	4	5
○	○	○	○	○

39. How dangerous do you think it is to smoke one or two cigarettes occasionally?

Not very dangerous				*Very dangerous*
1	2	3	4	5
○	○	○	○	○

40. How many young people your age out of 100 do you think smoke tobacco cigarettes?

____

41. Have you ever smoked a tobacco cigarette?

No ○ *Yes* ○

42. Have you ever tried tobacco cigarette smoking, even a few puffs?

No ○ *Yes* ○

The next questions ask about your knowledge and use of e-cigarettes. An e-cigarette is an electronic device that runs on a battery and produces vapour instead of smoke. There are many types of e-cigarettes and other vaping devices, such as e-hookah and vape pens, with some looking like regular cigarettes and some looking like pens. Some common brands are E-Lites, NJOY, Blu, and Nicolites.

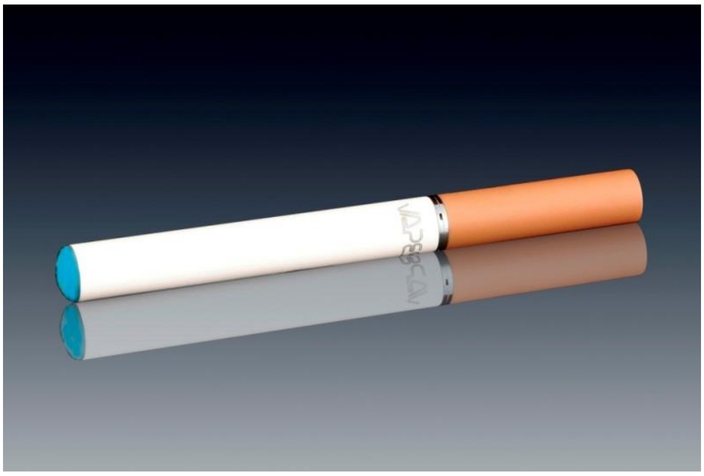

43. Please cross the circles for (a), (b) and (c) that best describe how you feel about using e-cigarettes:

a)

Unattractive				***Attractive***
**1**	**2**	**3**	**4**	**5**
◯	◯	◯	◯	◯

b)

Not cool				***Cool***
**1**	**2**	**3**	**4**	**5**
◯	◯	◯	◯	◯

c)

Boring				***Fun***
**1**	**2**	**3**	**4**	**5**
◯	◯	◯	◯	◯

44. Before today, have you ever heard of <u>e-cigarettes</u>?

No ○ *Yes* ○

45. How many young people your age out of 100 do you think use <u>e-cigarettes</u>?

____

46. Have you ever used an <u>e-cigarette</u>?

No ○ *Yes* ○

If you answered YES to question 46 please go to question 47. Otherwise, please skip the next question and go to question 48.

47. In the past 30 days, on how many days did you use an <u>e-cigarette</u>? __________

The next three questions ask for general information about you:

48. What is your gender?

Male ◯ *Female* ◯

49. How old are you? _____________ years

50. What is your ethnic group?

A. *White*
B. *Mixed/multiple ethnic groups*
C. *Asian/Asian British*
D. *Black/African/Caribbean/Black British*
E. *Other ethnic group*

Please check you have answered every question.

Thank you for your participation!

Health Group Booklet

"Study on e-cigarettes"

- Please fill in this form carefully.
- There are no right or wrong answers. We are interested only in what you think.
- It is important that the information you give is accurate and truthful.
- The researchers have no way of finding out who you are so you can be totally honest (please don't write your name on this survey).

Study details

An e-cigarette is an electronic device that runs on a battery and produces vapour instead of smoke.

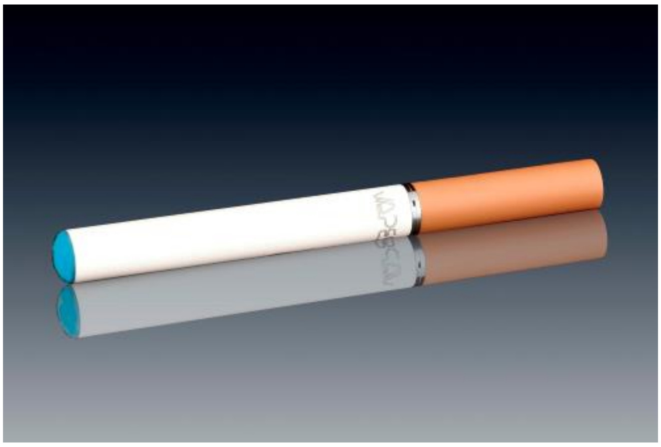

The aim of this study is to describe what people like you think about adverts for e-cigarettes.

What the study involves:

- You will be shown 10 e-cigarette adverts and asked to rate each one
- You will then be asked several questions about your thoughts on smoking tobacco cigarettes and using e-cigarettes
- You will also be asked several general questions about you
- For each question please cross the circle that best describes your views. For example, if you like the advert quite a lot you would cross the third circle as shown below:

How much do you like this advert (not the product)?

Not at all			*A lot*
1	2	3	4
○	○	⊗	○

Next, you will be shown 10 e-cigarette adverts and asked to rate each one.

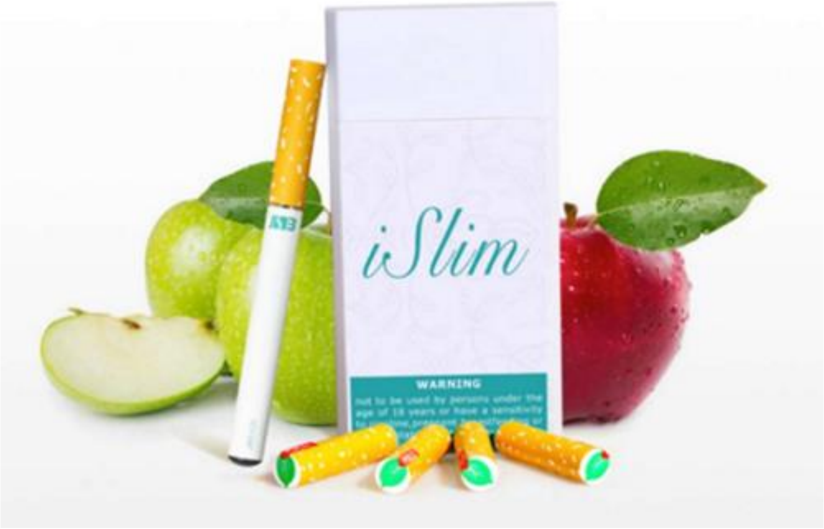

21. How much do you like this advert (not the product)?

Not at all			*A lot*
1	2	3	4
◯	◯	◯	◯

22. Does this advert make you want to buy and try this product?

Not at all			*Yes, a lot*
1	2	3	4
◯	◯	◯	◯

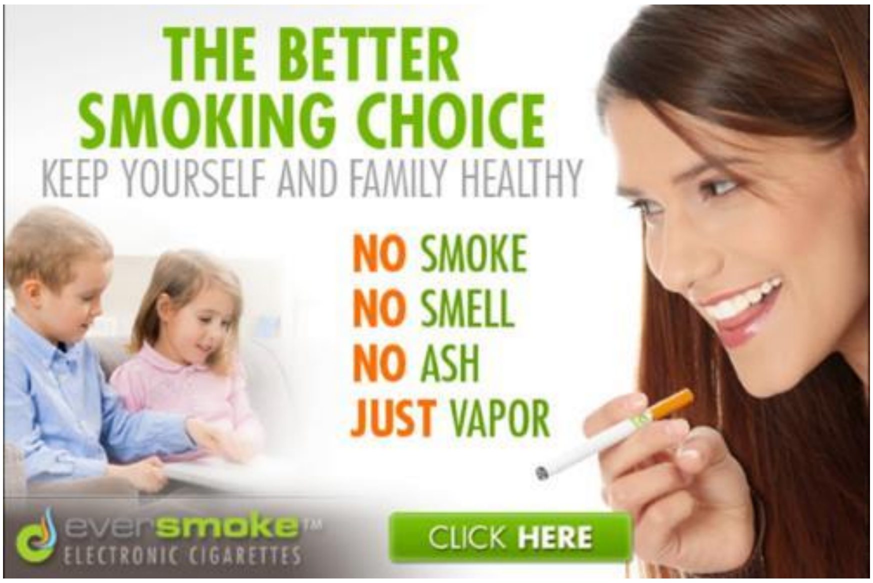

23. How much do you like this advert (not the product)?

Not at all			*A lot*
1	2	3	4
○	○	○	○

24. Does this advert make you want to buy and try this product?

Not at all			*Yes, a lot*
1	2	3	4
○	○	○	○

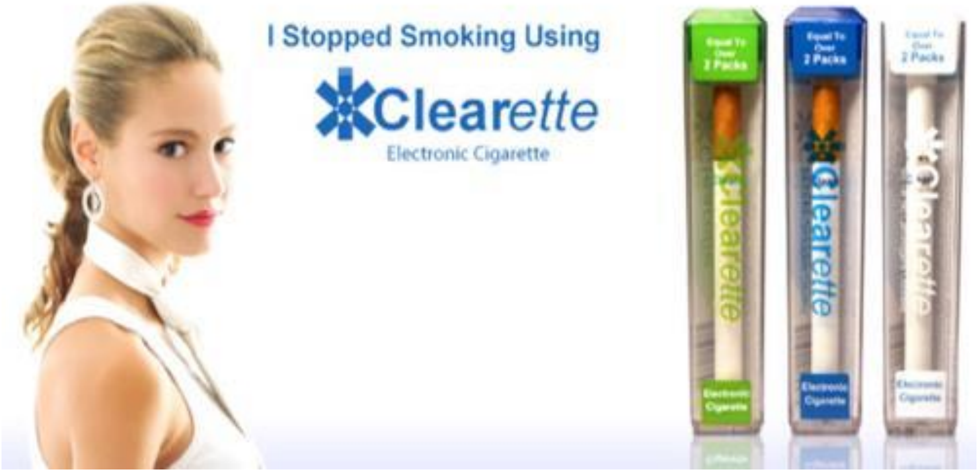

25. How much do you like this advert (not the product)?

Not at all			*A lot*
1	2	3	4
○	○	○	○

26. Does this advert make you want to buy and try this product?

Not at all			*Yes, a lot*
1	2	3	4
○	○	○	○

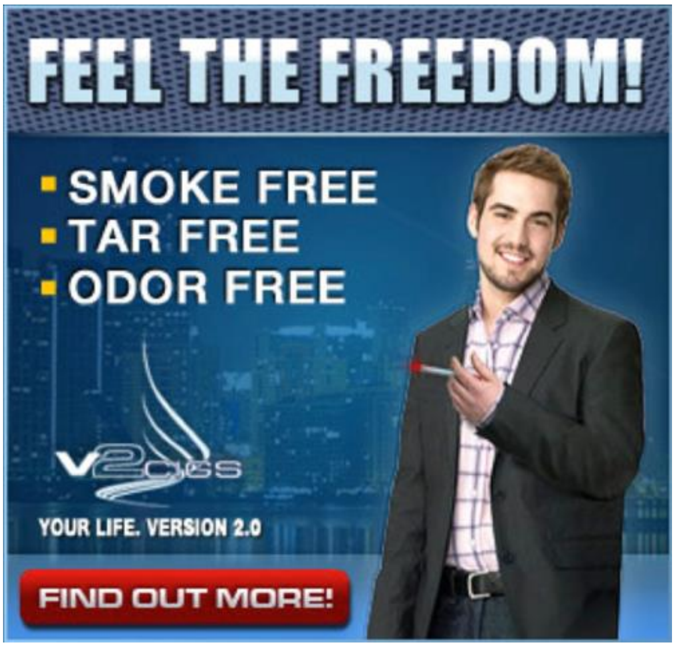

27. How much do you like this advert (not the product)?

Not at all			*A lot*
1	2	3	4
◯	◯	◯	◯

28. Does this advert make you want to buy and try this product?

Not at all			*Yes, a lot*
1	2	3	4
◯	◯	◯	◯

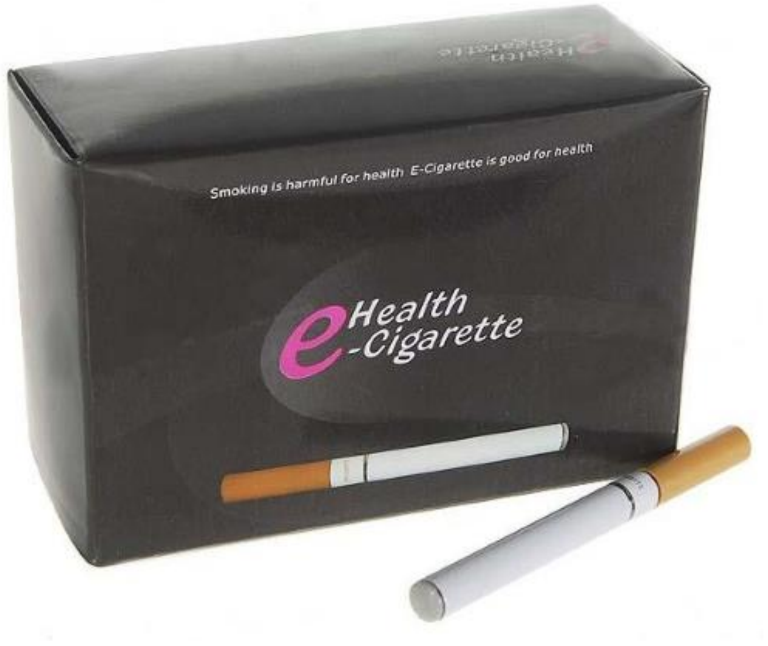

29. How much do you like this advert (not the product)?

Not at all			*A lot*
1	2	3	4
○	○	○	○

30. Does this advert make you want to buy and try this product?

Not at all			*Yes, a lot*
1	2	3	4
○	○	○	○

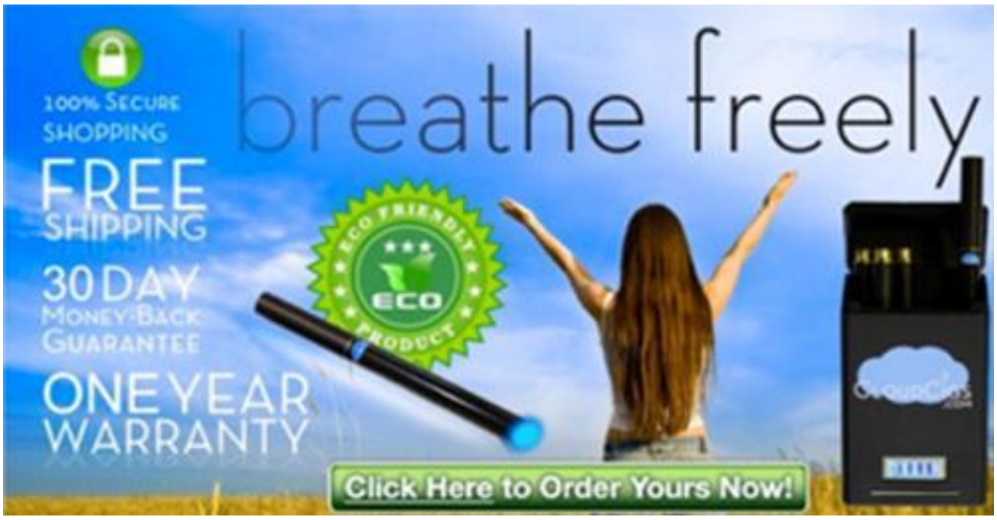

31. How much do you like this advert (not the product)?

Not at all			*A lot*
1	2	3	4
○	○	○	○

32. Does this advert make you want to buy and try this product?

Not at all			*Yes, a lot*
1	2	3	4
○	○	○	○

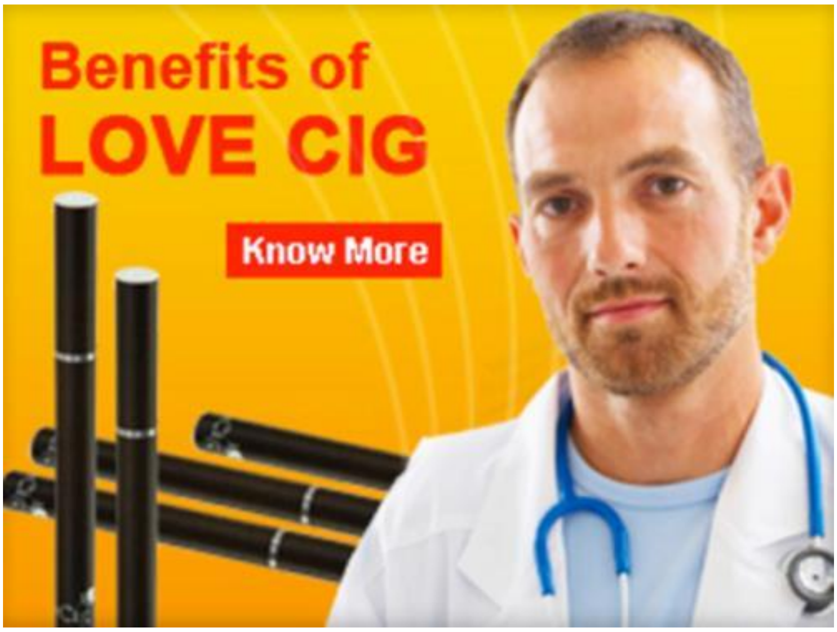

33. How much do you like this advert (not the product)?

Not at all			*A lot*
1	2	3	4
○	○	○	○

34. Does this advert make you want to buy and try this product?

Not at all			*Yes, a lot*
1	2	3	4
○	○	○	○

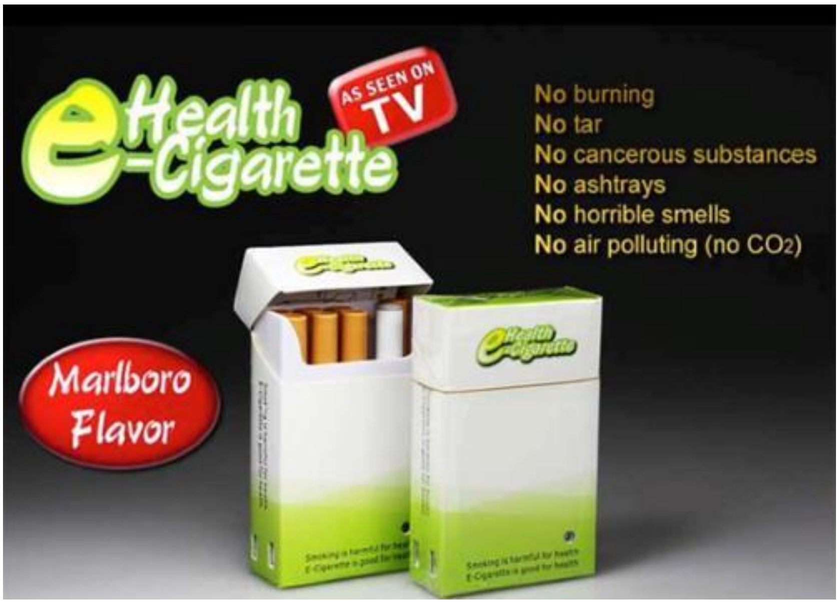

35. How much do you like this advert (not the product)?

Not at all			*A lot*
1	2	3	4
○	○	○	○

36. Does this advert make you want to buy and try this product?

Not at all			*Yes, a lot*
1	2	3	4
○	○	○	○

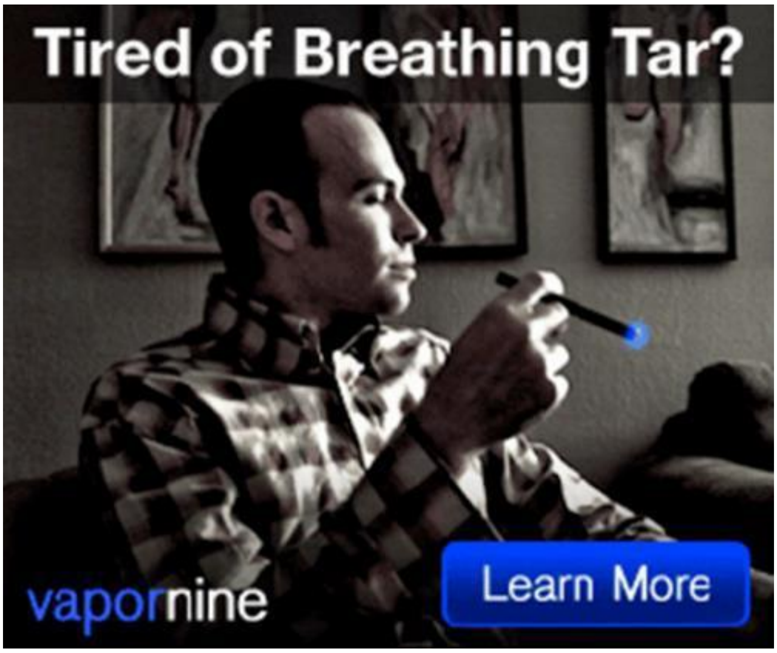

37. How much do you like this advert (not the product)?

Not at all			*A lot*
1	2	3	4
○	○	○	○

38. Does this advert make you want to buy and try this product?

Not at all			*Yes, a lot*
1	2	3	4
○	○	○	○

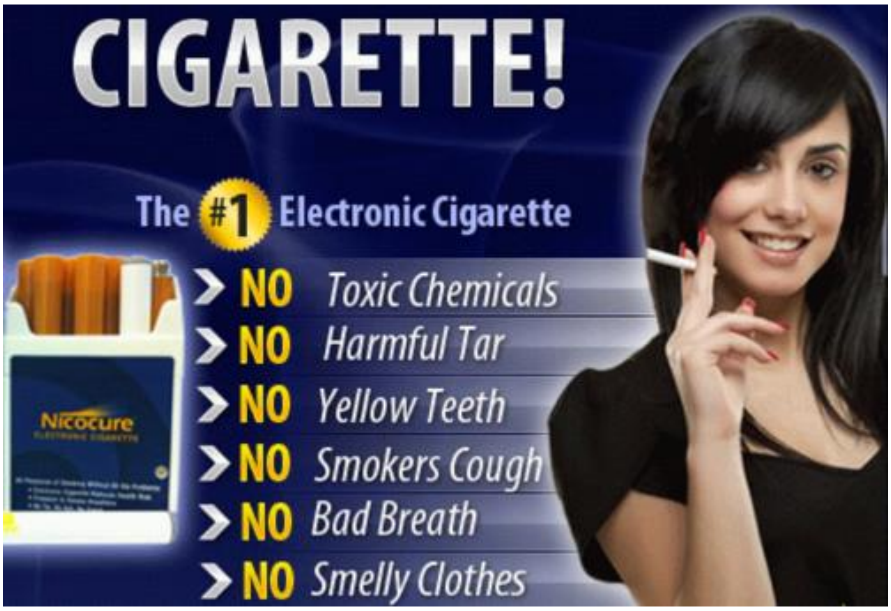

39. How much do you like this advert (not the product)?

Not at all			*A lot*
1	2	3	4
○	○	○	○

40. Does this advert make you want to buy and try this product?

Not at all			*Yes, a lot*
1	2	3	4
○	○	○	○

The next questions ask about <u>tobacco cigarettes</u>.

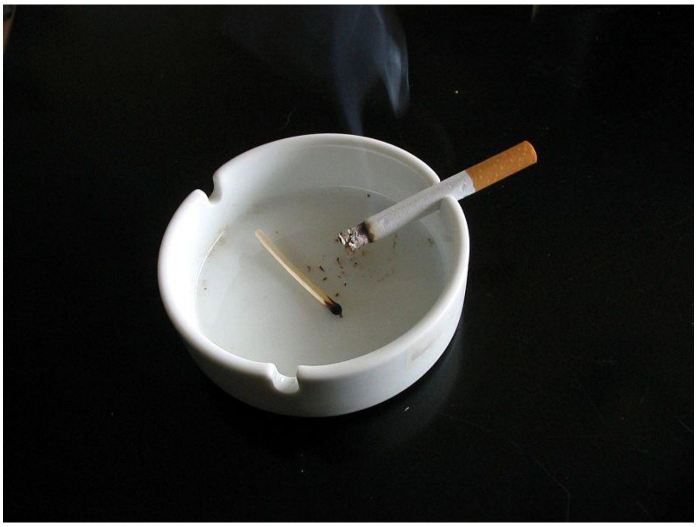

21. Please cross the circles for (a), (b) and (c) that best describe how you feel about smoking tobacco cigarettes:

a)

Unattractive				*Attractive*
1	2	3	4	5
○	○	○	○	○

b)

Not cool				*Cool*
1	2	3	4	5
○	○	○	○	○

c)

Boring				*Fun*
1	2	3	4	5
○	○	○	○	○

Please cross the circle that best describes your views on each of the next 12 statements:

22. Kids who smoke get more respect from others.

Strongly disagree	*Somewhat disagree*	*Neither agree nor disagree*	*Somewhat agree*	*Strongly agree*
1	2	3	4	5
○	○	○	○	○

23. Kids who smoke have more friends.

Strongly disagree	*Somewhat disagree*	*Neither agree nor disagree*	*Somewhat agree*	*Strongly agree*
1	2	3	4	5
○	○	○	○	○

24. Kids who smoke go out on more dates.

Strongly disagree	*Somewhat disagree*	*Neither agree nor disagree*	*Somewhat agree*	*Strongly agree*
1	2	3	4	5
○	○	○	○	○

25. Smoking helps people cope better with frustrations.

Strongly disagree	*Somewhat disagree*	*Neither agree nor disagree*	*Somewhat agree*	*Strongly agree*
1	2	3	4	5
○	○	○	○	○

26. Smoking is pleasurable.

Strongly disagree	*Somewhat disagree*	*Neither agree nor disagree*	*Somewhat agree*	*Strongly agree*
1	2	3	4	5
○	○	○	○	○

27. Smoking cigarettes relieves tension.

Strongly disagree	*Somewhat disagree*	*Neither agree nor disagree*	*Somewhat agree*	*Strongly agree*
1	2	3	4	5
◯	◯	◯	◯	◯

28. Smoking stinks.

Strongly disagree	*Somewhat disagree*	*Neither agree nor disagree*	*Somewhat agree*	*Strongly agree*
1	2	3	4	5
◯	◯	◯	◯	◯

29. Smoking is a messy habit.

Strongly disagree	*Somewhat disagree*	*Neither agree nor disagree*	*Somewhat agree*	*Strongly agree*
1	2	3	4	5
◯	◯	◯	◯	◯

30. Smoking makes teeth yellow.

Strongly disagree	*Somewhat disagree*	*Neither agree nor disagree*	*Somewhat agree*	*Strongly agree*
1	2	3	4	5
◯	◯	◯	◯	◯

31. Smoking can affect the health of others.

Strongly disagree	*Somewhat disagree*	*Neither agree nor disagree*	*Somewhat agree*	*Strongly agree*
1	2	3	4	5
◯	◯	◯	◯	◯

32. Smoking cigarettes is hazardous to people's health.

Strongly disagree	*Somewhat disagree*	*Neither agree nor disagree*	*Somewhat agree*	*Strongly agree*
1	2	3	4	5
◯	◯	◯	◯	◯

33. Cigarette smoke bothers other people.

Strongly disagree	*Somewhat disagree*	*Neither agree nor disagree*	*Somewhat agree*	*Strongly agree*
1	2	3	4	5
◯	◯	◯	◯	◯

The following 3 questions ask whether you might smoke tobacco cigarettes in the future.

34. Do you think you will smoke a tobacco cigarette at any time during the next year?

Definitely not	*Probably not*	*Probably yes*	*Definitely yes*
1	2	3	4
○	○	○	○

35. Do you think you will be smoking tobacco cigarettes when you are 18 years old?

Definitely not	*Probably not*	*Probably yes*	*Definitely yes*
1	2	3	4
○	○	○	○

36. If one of your friends offered you a tobacco cigarette, would you smoke it?

Definitely not	*Probably not*	*Probably yes*	*Definitely yes*
1	2	3	4
○	○	○	○

In the following three questions please cross the circles that best describe your opinions on smoking tobacco cigarettes.

37. Smoking can harm your health.

Strongly disagree				*Strongly agree*
1	2	3	4	5
○	○	○	○	○

38. How dangerous do you think it is to smoke more than 10 cigarettes a day?

Not very dangerous				*Very dangerous*
1	2	3	4	5
○	○	○	○	○

39. How dangerous do you think it is to smoke one or two cigarettes occasionally?

Not very dangerous				*Very dangerous*
1	2	3	4	5
○	○	○	○	○

40. How many young people your age out of 100 do you think smoke tobacco cigarettes?

____

41. Have you ever smoked a tobacco cigarette?

No ○ *Yes* ○

42. Have you ever tried tobacco cigarette smoking, even a few puffs?

No ○ *Yes* ○

The next questions ask about your knowledge and use of e-cigarettes. An e-cigarette is an electronic device that runs on a battery and produces vapour instead of smoke. There are many types of e-cigarettes and other vaping devices, such as e-hookah and vape pens, with some looking like regular cigarettes and some looking like pens. Some common brands are E-Lites, NJOY, Blu, and Nicolites.

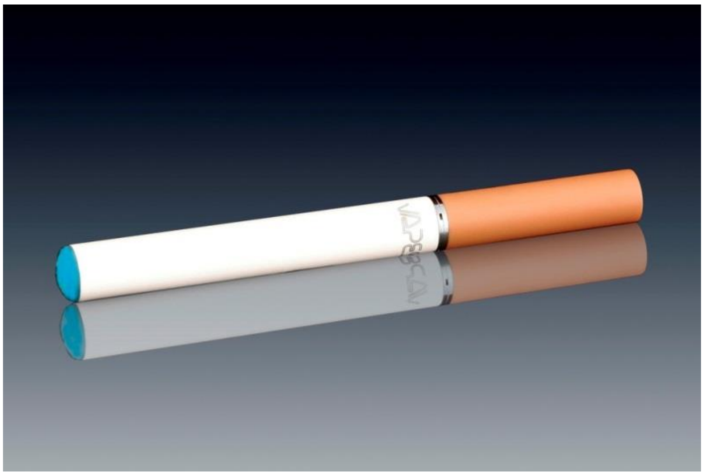

43. Please cross the circles for (a), (b) and (c) that best describe how you feel about using e-cigarettes:

a)

Unattractive				***Attractive***
**1**	**2**	**3**	**4**	**5**
○	○	○	○	○

b)

Not cool				***Cool***
**1**	**2**	**3**	**4**	**5**
○	○	○	○	○

c)

Boring				***Fun***
**1**	**2**	**3**	**4**	**5**
○	○	○	○	○

44. Before today, have you ever heard of <u>e-cigarettes</u>?

No ○ *Yes* ○

45. How many young people your age out of 100 do you think use <u>e-cigarettes</u>?

____

46. Have you ever used an <u>e-cigarette</u>?

No ○ *Yes* ○

If you answered YES to question 46 please go to question 47. Otherwise, please skip the next question and go to question 48.

47. In the past 30 days, on how many days did you use an <u>e-cigarette</u>? __________

The next three questions ask for general information about you:

48. What is your gender?

Male	*Female*
○	○

49. How old are you? _____________ years

50. What is your ethnic group?

F. *White*
G. *Mixed/multiple ethnic groups*
H. *Asian/Asian British*
I. *Black/African/Caribbean/Black British*
J. *Other ethnic group*

Please check you have answered every question.

Thank you for your participation!

Control Group Booklet

“Study on e-cigarettes”

- Please fill in this form carefully.
- There are no right or wrong answers. We are interested only in what you think.
- It is important that the information you give is accurate and truthful.
- The researchers have no way of finding out who you are so you can be totally honest (please don’t write your name on this survey).

Study details

An e-cigarette is an electronic device that runs on a battery and produces vapour instead of smoke.

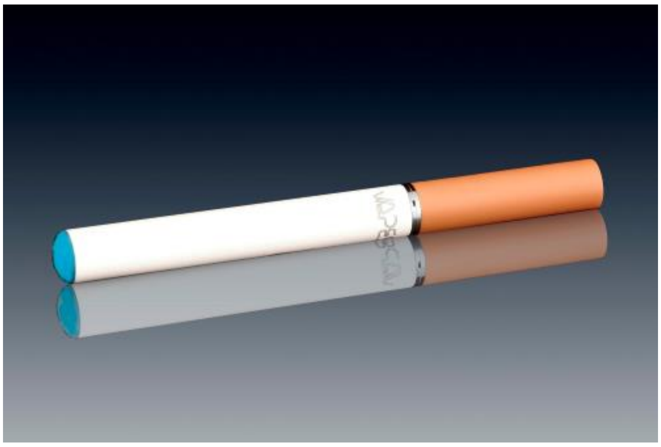

The aim of this study is to describe what people like you think about e-cigarettes.

What the study involves:

- You will be asked several questions about your thoughts on smoking tobacco cigarettes and using e-cigarettes

- For each question please cross the circle that best describes your views. For example, if you think that using e-cigarettes is moderately attractive you would respond by crossing the circle as shown below:

Please cross the circle that best describes how you feel about using e-cigarettes:

Unattractive				*Attractive*
1	2	3	4	5
○	○	○	☒	○

The next questions ask about <u>tobacco cigarettes</u>.

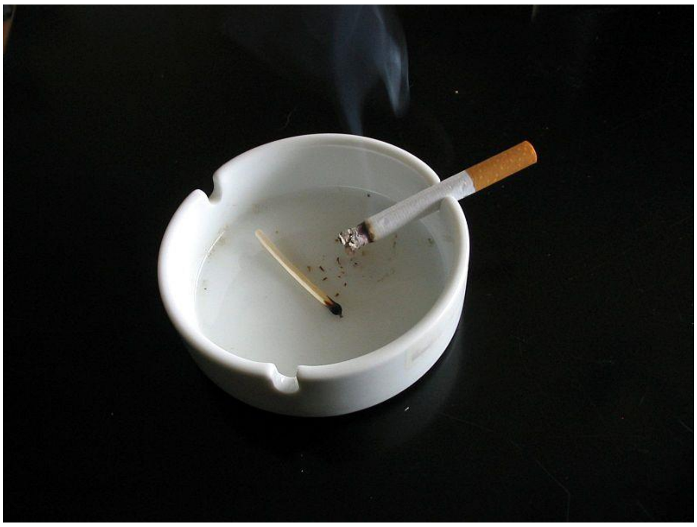

1. Please cross the circles for (a), (b) and (c) that best describe how you feel about smoking tobacco cigarettes:

a)

Unattractive				*Attractive*
1	2	3	4	5
○	○	○	○	○

b)

Not cool				*Cool*
1	2	3	4	5
○	○	○	○	○

c)

Boring				*Fun*
1	2	3	4	5
○	○	○	○	○

Please cross the circle that best describes your views on each of the next 12 statements:

2. Kids who smoke get more respect from others.

Strongly disagree	*Somewhat disagree*	*Neither agree nor disagree*	*Somewhat agree*	*Strongly agree*
1	2	3	4	5
○	○	○	○	○

3. Kids who smoke have more friends.

Strongly disagree	*Somewhat disagree*	*Neither agree nor disagree*	*Somewhat agree*	*Strongly agree*
1	2	3	4	5
○	○	○	○	○

4. Kids who smoke go out on more dates.

Strongly disagree	*Somewhat disagree*	*Neither agree nor disagree*	*Somewhat agree*	*Strongly agree*
1	2	3	4	5
○	○	○	○	○

5. Smoking helps people cope better with frustrations.

Strongly disagree	*Somewhat disagree*	*Neither agree nor disagree*	*Somewhat agree*	*Strongly agree*
1	2	3	4	5
○	○	○	○	○

6. Smoking is pleasurable.

Strongly disagree	*Somewhat disagree*	*Neither agree nor disagree*	*Somewhat agree*	*Strongly agree*
1	2	3	4	5

7. Smoking cigarettes relieves tension.

Strongly disagree	*Somewhat disagree*	*Neither agree nor disagree*	*Somewhat agree*	*Strongly agree*
1	2	3	4	5
○	○	○	○	○

8. Smoking stinks.

Strongly disagree	*Somewhat disagree*	*Neither agree nor disagree*	*Somewhat agree*	*Strongly agree*
1	2	3	4	5
○	○	○	○	○

9. Smoking is a messy habit.

Strongly disagree	*Somewhat disagree*	*Neither agree nor disagree*	*Somewhat agree*	*Strongly agree*
1	2	3	4	5
○	○	○	○	○

10. Smoking makes teeth yellow.

Strongly disagree	*Somewhat disagree*	*Neither agree nor disagree*	*Somewhat agree*	*Strongly agree*
1	2	3	4	5
○	○	○	○	○

11. Smoking can affect the health of others.

Strongly disagree	*Somewhat disagree*	*Neither agree nor disagree*	*Somewhat agree*	*Strongly agree*
1	2	3	4	5
○	○	○	○	○

12. Smoking cigarettes is hazardous to people's health.

Strongly disagree	*Somewhat disagree*	*Neither agree nor disagree*	*Somewhat agree*	*Strongly agree*
1	2	3	4	5
○	○	○	○	○

13. Cigarette smoke bothers other people.

Strongly disagree	*Somewhat disagree*	*Neither agree nor disagree*	*Somewhat agree*	*Strongly agree*
1	2	3	4	5
○	○	○	○	○

The following 3 questions ask whether you might smoke tobacco cigarettes in the future.

14. Do you think you will smoke a tobacco cigarette at any time during the next year?

Definitely not	*Probably not*	*Probably yes*	*Definitely yes*
1	2	3	4
○	○	○	○

15. Do you think you will be smoking tobacco cigarettes when you are 18 years old?

Definitely not	*Probably not*	*Probably yes*	*Definitely yes*
1	2	3	4
○	○	○	○

16. If one of your friends offered you a tobacco cigarette, would you smoke it?

Definitely not	*Probably not*	*Probably yes*	*Definitely yes*
1	2	3	4
○	○	○	○

In the following three questions please cross the circles that best describe your opinions on smoking <u>tobacco cigarettes</u>.

17. Smoking can harm your health.

Strongly disagree				*Strongly agree*
1	2	3	4	5
○	○	○	○	○

18. How dangerous do you think it is to smoke more than 10 cigarettes a day?

Not very dangerous				*Very dangerous*
1	2	3	4	5
○	○	○	○	○

19. How dangerous do you think it is to smoke one or two cigarettes occasionally?

Not very dangerous				*Very dangerous*
1	2	3	4	5
○	○	○	○	○

20. How many young people your age out of 100 do you think smoke tobacco cigarettes?

____

21. Have you ever smoked a tobacco cigarette?

No	*Yes*
◯	◯

22. Have you ever tried tobacco cigarette smoking, even a few puffs?

No	*Yes*
◯	◯

The next questions ask about your knowledge and use of e-cigarettes. An e-cigarette is an electronic device that runs on a battery and produces vapour instead of smoke. There are many types of e-cigarettes and other vaping devices, such as e-hookah and vape pens, with some looking like regular cigarettes and some looking like pens. Some common brands are E-Lites, NJOY, Blu, and Nicolites.

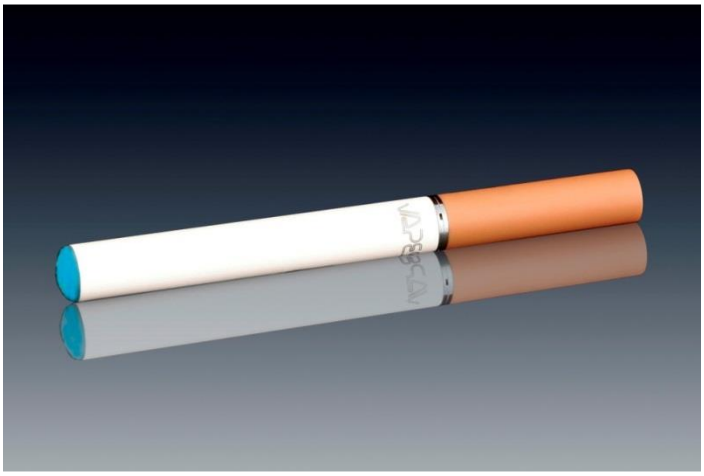

23. Please cross the circles for (a), (b) and (c) that best describe how you feel about using e-cigarettes:

a)

Unattractive				***Attractive***
**1**	**2**	**3**	**4**	**5**
○	○	○	○	○

b)

Not cool				***Cool***
**1**	**2**	**3**	**4**	**5**
○	○	○	○	○

c)

Boring				***Fun***
**1**	**2**	**3**	**4**	**5**
○	○	○	○	○

24. Before today, have you ever heard of <u>e-cigarettes</u>?

No ○ *Yes* ○

25. How many young people your age out of 100 do you think use <u>e-cigarettes</u>?

____

26. Have you ever used an <u>e-cigarette</u>?

No ○ *Yes* ○

If you answered YES to question 26 please go to question 27. Otherwise, please skip the next question and go to question 28.

27. In the past 30 days, on how many days did you use an <u>e-cigarette</u>? __________

The next three questions ask for general information about you:

28. What is your gender?

Male ○ *Female* ○

29. How old are you? _____________ years

30. What is your ethnic group?

K. White
L. Mixed/multiple ethnic groups
M. Asian/Asian British
N. Black/African/Caribbean/Black British
O. Other ethnic group

Please check you have answered every question.

Thank you for your participation!
